# Exploring metabolic candidates and inflammatory mediation in Parkinson’s disease: a pilot integrative metabolomics and Mendelian randomization study

**DOI:** 10.7717/peerj.21670

**Published:** 2026-09-28

**Authors:** Yue Lang, Hui Zhang, Rui Feng, Yongzhong Lin

**Affiliations:** Department of Neurology, The Second Affiliated Hospital of Dalian Medical University, Dalian, Liaoning, China

**Keywords:** Parkinson’s disease, Metabolomics, Mendelian randomization, Inflammatory cytokines, Caffeine metabolism, Mediation analysis

## Abstract

**Background:**

Parkinson’s disease (PD) is a complex neurodegenerative disorder characterized by multifaceted molecular dysregulation. Integrating genetic approaches with metabolomics may help to systematically investigate potential links between metabolites, inflammatory proteins, and PD risk.

**Methods:**

In this pilot discovery phase, untargeted Liquid Chromatography-Mass Spectrometry (LC-MS) was conducted in a small cohort (15 PD patients and 10 healthy controls). Differentially expressed metabolites (DEMs) were identified using variable importance in projection (VIP) > 1, |log_2_FC| ≥ 1, and false discovery rate (FDR)-adjusted *q*-values < 0.05 (Benjamini–Hochberg method), representing the metabolites remaining significant after multiple-testing correction applied directly at the metabolite discovery stage. Prioritized metabolites were selected by Random Forest, Least absolute shrinkage and selection operator (LASSO) regression, and Kyoto Encyclopedia of Genes and Genomes (KEGG) pathway enrichment. Two-sample Mendelian randomization (MR) was performed using PD Genome-Wide Association Study (GWAS) data from FinnGen R12 (European ancestry: 5,861 cases, 494,487 controls) to evaluate putative causal associations, with FDR correction applied separately within the metabolite and inflammatory protein exposure sets. Mediation MR was used to explore potential mediating roles of 91 circulating inflammatory proteins.

**Results:**

A total of 3,537 metabolites were identified, of which 570 were classified as DEMs, representing the features remaining significant after multiple-testing correction. Integration of Random Forest and LASSO identified 3-phenylpropionylglycine as a key candidate metabolite. KEGG analysis highlighted caffeine metabolism as the top enriched pathway. After FDR correction, MR analyses suggested inverse associations between genetically predicted levels of 3-phenylpropionylglycine (*P_FDR_* = 0.0077, OR = 0.82) and 7-methylxanthine (*P_FDR_* = 0.0349, OR = 0.83) and PD risk. Mediation analysis further indicated that SULT1A1 may partially mediate the association between 3-phenylpropionylglycine and PD.

**Conclusion:**

This exploratory study integrates pilot-scale metabolomics with genetic analyses to identify candidate metabolic signals and inflammatory pathways linked to PD. However, given the small metabolomics sample size, cross-cohort data integration, and potential biological mismatch between plasma-derived metabolites and genetically predicted metabolite levels, these findings should be considered hypothesis-generating and interpreted with caution.

## Introduction

Parkinson’s disease (PD) is a common progressive neurodegenerative disorder, clinically characterized by bradykinesia, resting tremor, rigidity, and postural instability. With the global aging population, the prevalence of PD continues to rise, imposing a substantial burden on public health worldwide (Bloem, Okun & Klein, 2021; Rocha et al., 2025). It is generally recognized that the core pathogenic mechanisms involve the loss of dopaminergic neurons in the substantia nigra–striatal pathway and abnormal aggregation of α-synuclein (Jiang et al., 2025). However, the precise etiology of PD remains incompletely understood. Accumulating evidence suggests that its development is influenced by a combination of aging, genetic predisposition, environmental exposures, and metabolic dysregulation (Soni & Shah, 2022; Hao et al., 2025; Gu et al., 2025). In this context, identifying robust molecular signals associated with disease risk remains an important research goal.

In recent years, the rapid advancement of metabolomics technologies has significantly propelled disease research, providing powerful tools for elucidating pathophysiological mechanisms and identifying novel candidate signals. These approaches have been widely applied across various conditions, including glioblastoma, non-small cell lung cancer, Alzheimer’s disease, and type 2 diabetes (Wang et al., 2025b, 2025c; Mai et al., 2025), yielding notable achievements in uncovering disease-specific metabolic pathways, constructing molecular subtypes, and identifying potential diagnostic and prognostic indicators. However, compared with other disease areas, metabolomics studies in PD remain relatively limited. Systematic analysis of the metabolic profiles of PD patients can comprehensively reflect disruptions in key processes such as energy metabolism, oxidative stress, lipid homeostasis, and neurotransmitter synthesis and degradation, thereby providing new perspectives and technical avenues for understanding PD molecular pathogenesis, defining molecular subtypes, and developing individualized intervention strategies (Kaleta et al., 2025).

Meanwhile, circulating inflammatory proteins are recognized as an additional critical contributor in the onset and progression of PD, driving disease processes by inducing neuroinflammation and promoting neuronal damage. However, their causal relationship with PD risk has yet to be clearly established (Frick et al., 2025; Yu et al., 2025). Therefore, systematically assessing the potential causal links between metabolomic profiles and inflammatory proteins from a genetic perspective may help generate hypotheses regarding the multidimensional regulatory network underlying PD.

Mendelian randomization (MR) is an innovative epidemiological approach that leverages single nucleotide polymorphisms (SNPs) as genetic instruments to mimic the randomization process in controlled trials, thereby enabling the investigation of causal relationships between exposures and disease outcomes (Wang et al., 2025a). Its inherent advantages include natural randomization, minimizing reverse causation, and reducing confounding (Sun et al., 2025).

While metabolomic alterations and neuroinflammation are both hallmarks of PD, the relationship between these two domains remains incompletely understood. In this study, we implemented a two-step Mendelian randomization framework integrating preliminary untargeted metabolomics findings with large-scale Genome-Wide Association Study (GWAS) data to explore potential causal relationships between circulating metabolites and PD risk. Recognizing the exploratory nature of the metabolomics discovery stage and the complexity of cross-cohort data integration, our aim was to investigate whether genetically predicted metabolite-associated signals may be related to PD susceptibility. In addition, we assessed whether circulating inflammatory proteins may act as potential mediating factors using a two-step MR framework involving 91 inflammatory cytokines. This analysis provides hypothesis-generating insights into possible metabolite–immune interactions in PD.

## Materials and Methods

### Study design

This study implemented an exploratory integrative framework combining pilot-scale clinical metabolomics with two-sample MR to identify potential causal metabolic signals for PD (Fig. 1). Untargeted metabolomic profiling of plasma samples from PD patients and healthy controls (HCs) was performed, and differentially expressed metabolites (DEMs) were identified following multiple-testing correction. Core metabolic features were prioritized using machine learning (Random Forest and least absolute shrinkage and selection operator) analysis and Kyoto Encyclopedia of Genes and Genomes (KEGG) pathway enrichment. These prioritized metabolites were then used as exposures in a two-sample MR framework, leveraging large-scale publicly available GWAS datasets to evaluate their potential putative causal association on PD risk. SNPs were selected as instrumental variables (IVs) and were required to satisfy three key assumptions: (1) relevance—the IVs are strongly associated with the exposure (typically indicated by an F-statistic > 10); (2) independence—the IVs are not associated with any known or unknown confounders; and (3) exclusion restriction—the IVs affect the outcome only through the exposure and not *via* alternative pathways. In addition, a mediation MR analysis involving 91 circulating inflammatory proteins was conducted to investigate their potential mechanistic role in the metabolite–PD axis.

**Figure 1:**
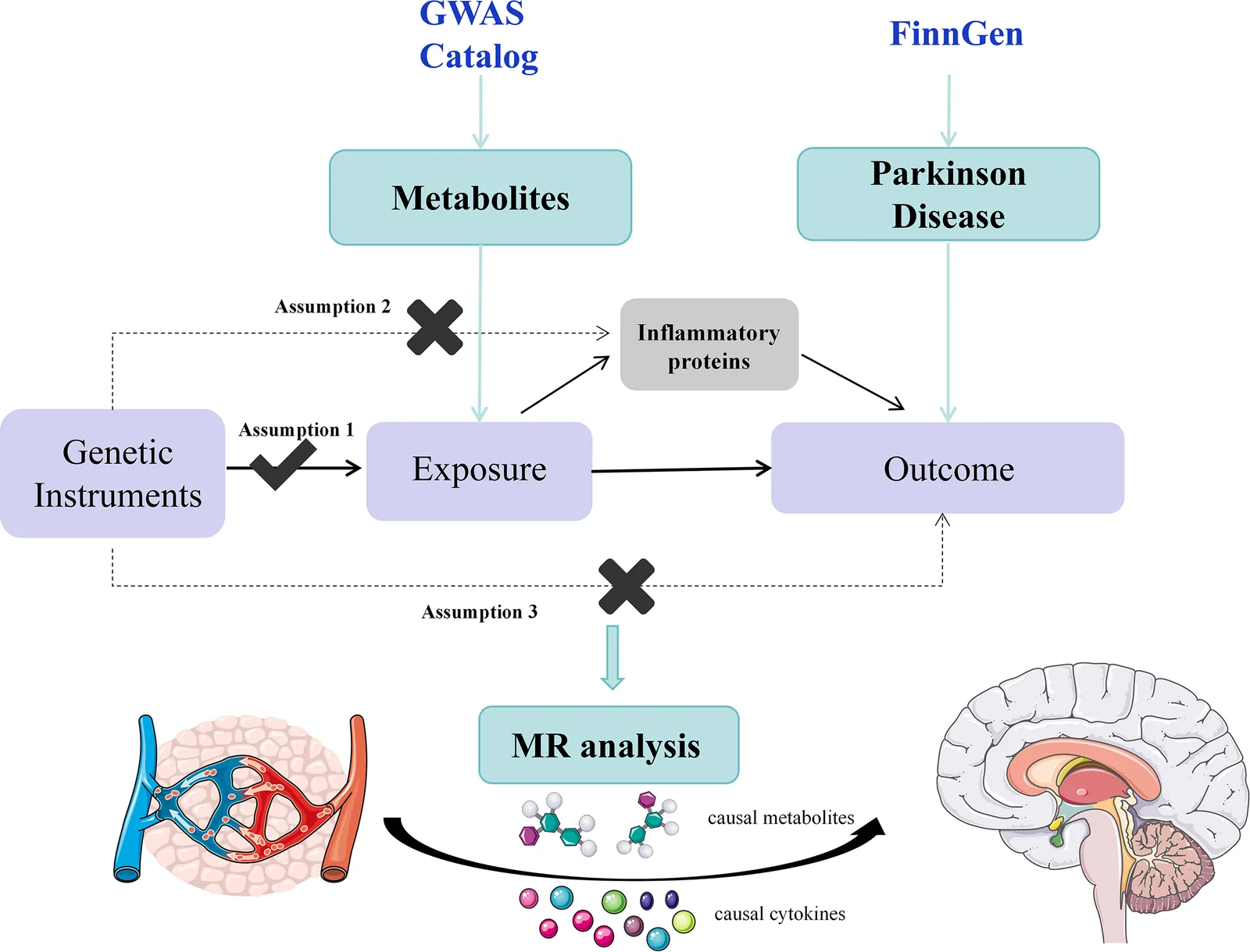
Flowchart of the study design.

### Data sources

#### Metabolomics data

This study was approved by the Ethics Committee of the Second Affiliated Hospital of Dalian Medical University (Approval No. KY2025-468-01). Between August 2025 and September 2025, patients were recruited from the Department of Neurology at the same hospital. Written informed consent was obtained from all participants prior to enrollment. Participants were rigorously screened based on the following predefined criteria:

Inclusion criteria were as follows:

(1) Patients diagnosed with Parkinson’s disease according to the Movement Disorder Society (MDS) clinical diagnostic criteria.
(2) Age between 40 and 85 years.
(3) Body mass index (BMI) between 18.5 and 30 kg/m^2^.
(4) Voluntary participation with written informed consent.

Exclusion criteria included:

(1) Secondary or atypical parkinsonism (*e.g*., drug-induced parkinsonism, vascular parkinsonism, or multiple system atrophy).
(2) Presence of other severe neurological disorders (*e.g*., vestibular system diseases, stroke, epilepsy, encephalitis, or history of traumatic brain injury) or psychiatric disorders (*e.g*., major depressive disorder or schizophrenia).
(3) Severe dysfunction of major organs, including heart, lung, liver, or kidney, or other serious systemic diseases.
(4) Diagnosed diabetes mellitus (particularly poorly controlled diabetes), thyroid dysfunction, or hyperlipidemia requiring statin therapy.
(5) Heavy smoking (>10 cigarettes per day) or alcohol consumption (>20 g/day) within the past year, or other substance dependence.

After screening, 25 independent whole-blood samples were included in this study, comprising 15 patients with PD and 10 HCs. Detailed demographic and clinical characteristics are presented in Table 1. Information regarding clinical anti-parkinsonian or dopaminergic medication status at the time of sampling was unavailable for analysis. Plasma from blood was centrifuged for 10 min at 3,000 rpm and was collected after an overnight fast and immediately stored at −80 °C until subsequent metabolomic analysis.

**Table 1:** General demographic data.

Variables	Total (*n* = 25)	HCs (*n* = 10)	PD (*n* = 15)
Age (years), Mean ± SD	62.52 ± 5.96	60.70 ± 6.91	63.73 ± 5.12
MMSE, Mean ± SD	26.92 ± 2.89	28.20 ± 0.79	26.07 ± 3.45
BMI, Mean ± SD	21.98 ± 2.04	22.15 ± 1.93	21.87 ± 2.18
Sex, *n* (%)			
Female	11 (44.00)	4 (40.00)	7 (46.67)
Male	14 (56.00)	6 (60.00)	8 (53.33)

**Note:**

SD, standard deviation; MMSE, mini-mental state examination; BMI, body mass index.

#### GWAS data

Exposures and mediators for the MR analysis were obtained from the GWAS Catalog (Cerezo et al., 2025) and were primarily derived from cerebrospinal fluid (CSF) metabolite levels (Wang et al., 2024), reflecting the genetic architecture of brain metabolites and their colocalization with human traits. For one metabolite lacking available CSF or European-ancestry data, summary statistics from an African-ancestry cohort were used as a single exception. PD was used as the outcome variable. GWAS summary data for PD were obtained from the FinnGen R12 release (Kurki et al., 2023), which included 5,861 cases and 494,487 controls, with a total sample size of 500,348 participants of European ancestry. Detailed participant information is provided in Table S1.

### Liquid chromatography-mass spectrometry-based metabolomics analysis

Blood samples were thawed on ice and 100 μL of each sample was mixed with 400 μL cold protein precipitation solvent (methanol:acetonitrile, 2:1) containing 4 μg/mL internal standards. After 1 min vortexing and 10 min ultrasonication on ice, samples were incubated overnight at −40 °C and centrifuged at 12,000 rpm for 20 min at 4 °C. Supernatants (150 μL) were transferred to liquid chromatography-mass spectrometry (LC-MS) vials for analysis. Quality control (QC) samples were prepared by pooling equal volumes of all extracts, injected five times before analysis for system equilibration, and after every ten samples to monitor batch stability.

LC-MS analysis was performed using a Waters ACQUITY UPLC I-Class Plus system coupled to a Thermo Q Exactive HF high-resolution mass spectrometer (Thermo Fisher Scientific, Waltham, MA, USA). Chromatographic separation was achieved on an ACQUITY UPLC HSS T3 column (100 × 2.1 mm, 1.8 μm) at 45 °C. Mobile phase A consisted of 0.1% formic acid in water, and mobile phase B was acetonitrile, with a flow rate of 0.35 mL/min and a total runtime of 16 min. Mass spectrometry (MS) was performed in both positive and negative electrospray ionization (ESI) modes using a Full MS/dd-MS^2^ acquisition method. The MS1 mass range was m/z 70–1,050, with MS1 and MS2 resolutions set at 60,000 and 15,000 FWHM, respectively, and stepped normalized collision energies (NCE) of 10, 20, and 40 eV.

### Metabolite identification

Raw MS data were converted to mzXML format using ProteoWizard (v3.0.8789; ProteoWizard software Foundation, Palo Alto, CA, USA) and processed in Progenesis QI (v3.0; Nonlinear Dynamics, London, UK) for baseline correction, peak detection, retention time alignment, peak area integration, and global ion intensity normalization to generate a feature matrix. Metabolite annotation was performed based on retention time, accurate mass, MS/MS fragmentation, and isotopic patterns, with reference to HMDB, LipidMaps, METLIN, and LuMet-Animal 3.0 databases.

### Multivariate statistical analysis

To evaluate overall differences in metabolomic profiles between PD patients and healthy controls, unsupervised Principal Component Analysis (PCA) was performed to assess sample distribution, experimental variability, and data quality. Supervised Partial Least Squares Discriminant Analysis (PLS-DA) and Orthogonal PLS-DA (OPLS-DA) were then applied to model group separation and identify discriminative metabolic features. Model robustness was assessed using seven-fold cross-validation and 200 permutation tests, and variable contributions were quantified by Variable Importance in Projection (VIP) scores.

### Differential metabolite selection

Metabolites with VIP > 1 derived from the OPLS-DA model were considered as candidate metabolites. These candidates were subsequently tested using the Mann–Whitney U test, and the resulting *P*-values were adjusted for multiple testing across all tested metabolites using the Benjamini–Hochberg false discovery rate (FDR) method. Metabolites were explicitly defined as significantly differentially expressed if they met both criteria of FDR-adjusted *q*-value < 0.05 and fold change (FC) ≥ 2.0 or ≤ 0.5, thereby ensuring that the identified cohort represents only the features remaining significant after multiple-testing correction.

### Metabolic pathway enrichment analysis

To investigate the biological relevance of the identified DEMs, pathway enrichment analysis was performed using the KEGG database. DEMs were mapped to KEGG pathways, and enrichment was assessed using the hypergeometric test. Resulting *P*-values were adjusted for multiple testing across all tested pathways using the Benjamini–Hochberg FDR method, and pathways with FDR-adjusted *q*-values < 0.05 were considered significantly enriched.

### Key DEMs

To prioritize metabolites for downstream MR analysis, two complementary strategies were applied. First, DEMs were subjected to feature selection using Random Forest and least absolute shrinkage and selection operator (LASSO) regression models, and metabolites consistently selected by both methods were retained. Second, metabolites involved in the top-ranked KEGG-enriched pathway were identified and intersected with the DEMs. The union of metabolites obtained from these two approaches was defined as prioritized candidate metabolites and used as exposures in subsequent MR analyses. Given the limited sample size of the metabolomics dataset, this feature selection process was considered exploratory. In addition, feature selection methods such as Random Forest may be sensitive to small sample sizes, and the results should be interpreted with caution.

### Selection of instrumental variables for MR

To minimize potential confounding and ensure robust IVs, we applied the following selection criteria based on previous studies:

(1) Selection of SNPs based on GWAS *P*-value: For exposures, SNPs were initially selected at genome-wide significance (*P* < 1 × 10^−8^). Because most exposure-related SNPs had an explanatory variance (R^2^) < 1% under this threshold, a more relaxed threshold (*P* < 1 × 10^−6^) was adopted to ensure an adequate number of SNPs. For circulating inflammatory proteins, a threshold of *P* < 1 × 10^−5^ was used, consistent with previous large-scale protein GWAS studies.
(2) Linkage disequilibrium (LD) Pruning: To ensure independence and reduce potential bias from LD, SNPs were pruned within a 10,000 kb window using an LD threshold of r^2^ < 0.001. Notably, L-aspartic acid was excluded from the downstream MR analysis because it failed to yield a sufficient number of independent SNPs after LD pruning in the referenced dataset.
(3) Instrument Strength and Harmonization: The strength of each SNP was evaluated using the F-statistic (F = β^2^/SE^2^), and those with F < 10 were excluded to avoid weak instrument bias. SNP harmonization was implemented using the harmonise_data (exposure_dat, outcome_dat, action = 2) function to flip inconsistent alleles and remove unalignable palindromic SNPs with intermediate allele frequencies, ensuring the directionality of the effect estimates. We recorded the number of SNP instruments for each exposure, as well as their corresponding F-statistics and variance explained (R^2^).
(4) pQTL Definition for Inflammatory Proteins: For inflammatory proteins, we used pQTLs identified including both cis- and trans-pQTL instruments based on data availability. Specifically, SNPs within ±1 Mb of the encoding gene were defined as cis-pQTLs, while more distant or trans-chromosomal SNPs were defined as trans-pQTLs. Cis-pQTLs were prioritized to reduce potential horizontal pleiotropy, and potential pleiotropic effects were further considered in sensitivity analyses.

### MR analysis

To evaluate the potential causal relationships between prioritized metabolites, inflammatory proteins, and PD, two-sample MR analyses were performed. For exposures with a single IV, putative causal associations were estimated using the Wald ratio. For those with multiple IVs, the inverse variance weighted (IVW) method was used as the primary analysis, supplemented by MR-Egger regression, weighted median, simple mode, and weighted mode analyses. The intercept from MR-Egger was used to assess horizontal pleiotropy. Results are reported as odds ratios (ORs) with 95% confidence intervals (CIs). To control for multiple testing, the Benjamini–Hochberg FDR correction was applied separately within predefined exposure families. Specifically, all metabolite–PD MR associations were corrected as one family, and all inflammatory protein–PD MR associations were corrected as a second independent family. Within each family, FDR adjustment was performed across all tested exposure–outcome associations in a unified manner. Associations with FDR-adjusted *q*-values < 0.05 were considered statistically significant. The validity of MR estimates relies on the assumptions of relevance, independence, and exclusion restriction, which cannot be fully verified in practice and should be considered when interpreting the findings.

### Mediation analysis

Two-step MR analyses were performed to investigate potential causal mediation effects. In the statistical model, the total effect of prioritized candidate metabolites (exposures) on PD (outcome) was decomposed into direct effects and indirect effects mediated through circulating inflammatory proteins (potential mediators). The effects of exposures on mediators and the effects of exposures on PD were each estimated using independent genetic instruments. Indirect effects, direct effects, and the proportion mediated were calculated using the following formulas:

${\beta _{indirect\; effect}} = {\beta _{exposure \to mediator}} \times {\beta _{mediator \to outcome}}$

${\beta _{direct\; effect}} = {\beta _{exposure \to outcome}} - \left( {{\beta _{exposure \to mediator}} \times {\beta _{mediator \to outcome}}} \right)$

${\beta _{mediation}} = \displaystyle{{{\beta _{indirect\; effect}}} \over {{\beta _{exposure \to outcome}}}} \times 100 \rm % .$

Confidence intervals for mediation effects were estimated using the Delta method. This mediation framework relies on several assumptions, including the absence of unmeasured confounding between the exposure, mediator, and outcome, as well as no horizontal pleiotropy affecting the mediator–outcome relationship. Given these assumptions, the mediation results should be interpreted cautiously and considered hypothesis-generating.

### Sensitivity analysis

To assess the robustness of the MR findings, comprehensive sensitivity analyses were performed. Heterogeneity among IVs was evaluated using Cochran’s Q statistic for both IVW and MR-Egger methods. Horizontal pleiotropy was assessed using the MR-Egger intercept test. MR-PRESSO global tests, including outlier and distortion tests, were applied to identify and correct potential outlier SNPs arising from pleiotropy. Leave-one-out analyses were conducted to determine whether causal estimates were driven by any single influential SNP. Results were visualized using scatter plots, funnel plots, and single-SNP forest plots.

All statistical analyses were performed using R software (version 4.4.1; R Core Team, 2024). Two-sample MR analyses were primarily conducted using the “TwoSampleMR” R package.

## Results

### Quality control of QC samples

To ensure the accuracy of metabolite detection and the stability of the acquired data, QC samples were included before and after sample analysis to systematically evaluate the performance of the mass spectrometry platform throughout the experimental process. PCA was first employed to reduce dimensionality and visualize overall data variability. The results showed that QC samples clustered tightly in the PCA score plot (Fig. 2A), indicating stable instrument performance and high reproducibility. Subsequently, hierarchical clustering based on Euclidean distance was performed, and the resulting dendrogram (Fig. 2B) demonstrated consistent clustering patterns among QC samples, with highly similar expression profiles, further confirming the system’s reliability and reproducibility. Pearson correlation analysis was then conducted among QC samples, and the corresponding heatmap (Fig. 2C) revealed high inter-sample correlation, reflecting stable signal output and controlled experimental variability. Finally, box plots of metabolite intensities across all samples were generated (Fig. 2D). QC samples exhibited uniform box heights and narrow distribution ranges, supporting the overall technical repeatability and intra-group consistency of the metabolomics dataset.

**Figure 2:**
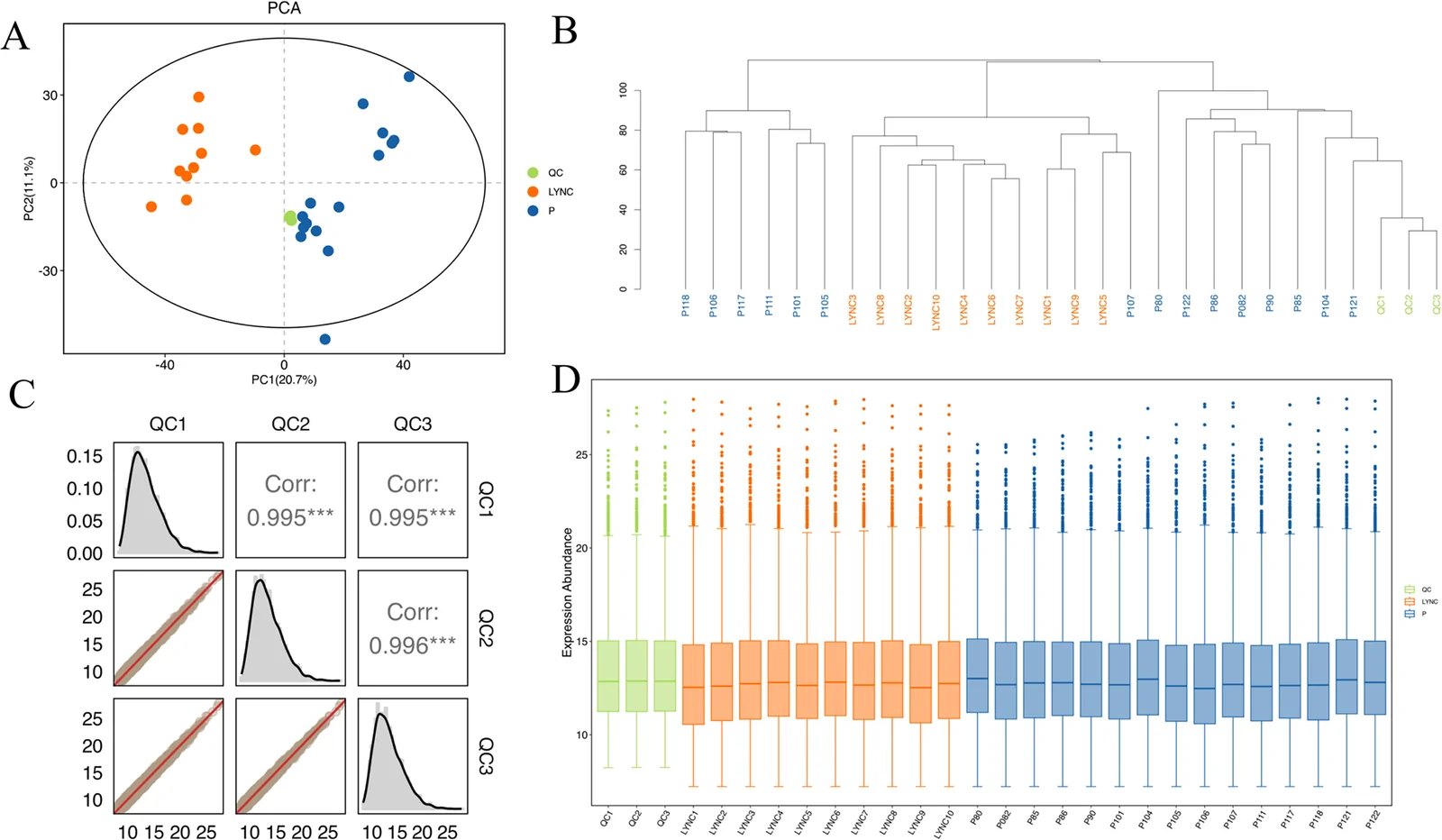
QC assessment of metabolomics data. (A) PCA score plot showing sample distribution. (B) Hierarchical clustering dendrogram of sample Euclidean distance. (C) Pairwise Pearson correlation matrix of QC samples. High correlation coefficients with *** (*p* < 0.001) demonstrate the high reproducibility and stability of the analytical platform. (D) Box plot of normalized metabolite intensities across samples.

### Metabolomic profiling of plasma samples

In this study, LC-MS-based metabolomic profiling of 25 plasma samples identified a total of 3,537 metabolites. Among them, 1,372 metabolites were detected in positive ion mode and 2,165 in negative ion mode. Lipids and lipid-like molecules, along with organic acids and their derivatives, were the predominant classes in both modes—accounting for 583 and 237 metabolites in positive mode, and 930 and 326 in negative mode, respectively.

### Multivariate statistical analysis

To investigate the global metabolic differences between PD patients and healthy controls, multivariate statistical analyses were conducted based on the metabolomic profiles of plasma samples. Unsupervised PCA (Fig. 3A) revealed a clear separation between the two groups without apparent outliers, indicating high data quality, stability, and strong intergroup discrimination. OPLS-DA (Fig. 3B) further demonstrated distinct metabolic patterns between PD and control groups, suggesting substantial metabolic alterations associated with PD. The corresponding loading plot (Fig. 3C) identified metabolites contributing most to group discrimination, while the S-plot (Fig. 3D) visualized both variable importance and correlation, highlighting key discriminatory metabolites. Model robustness was confirmed by 200-permutation testing (Fig. 3E). The original OPLS-DA model exhibited excellent cumulative explanatory power and predictive capability (R^2^Y = 0.939, Q^2^ = 0.912). Crucially, the regression lines retained an R^2^ intercept of 0.939 and a Q^2^ intercept of −0.321. The negative Q^2^ intercept (which is well below 0.05) strictly demonstrates that the primary OPLS-DA model is robust, reliable, and entirely free of overfitting.

**Figure 3:**
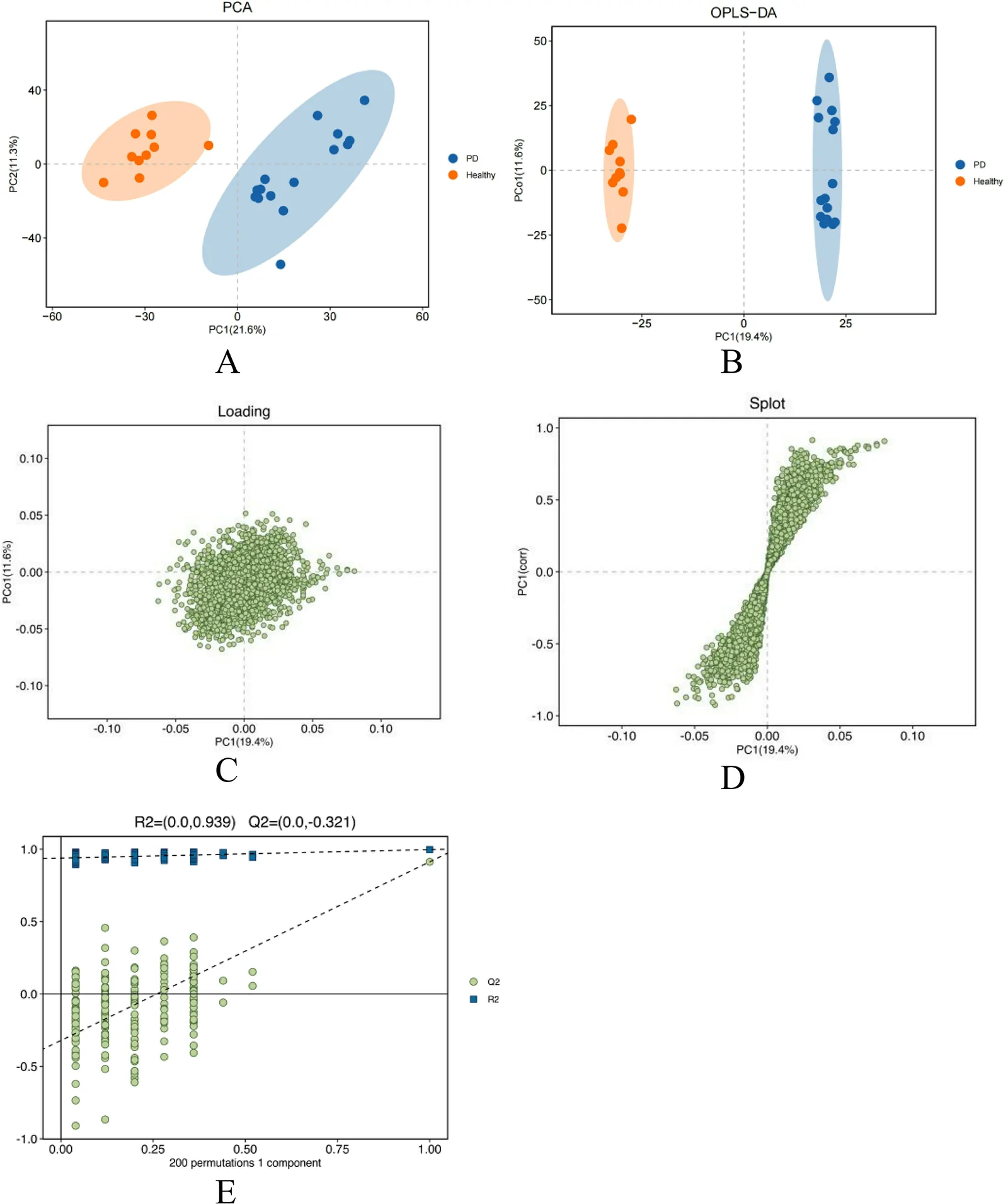
(A) PCA score plot. (B) OPLS-DA score plot. (C) OPLS-DA loading plot. (D) S-plot derived from OPLS-DA. (E) Permutation test plot.

### Identification of differential metabolites

To identify PD-related metabolic alterations, out of the 3,537 identified features, metabolites with VIP scores > 1 were extracted from the OPLS-DA model, yielding 1,014 candidate metabolites. Subsequent group comparisons using the criteria of FDR-adjusted *q*-value < 0.05 and FC ≥ 2.0 or ≤ 0.5 identified exactly 570 significantly DEMs remaining significant after multiple-testing correction, including 263 upregulated and 307 downregulated metabolites (Fig. 4A). To further illustrate the relationships among samples and the expression patterns of these metabolites, a heatmap was generated based on the top 50 DEMs (Fig. 4B).

**Figure 4:**
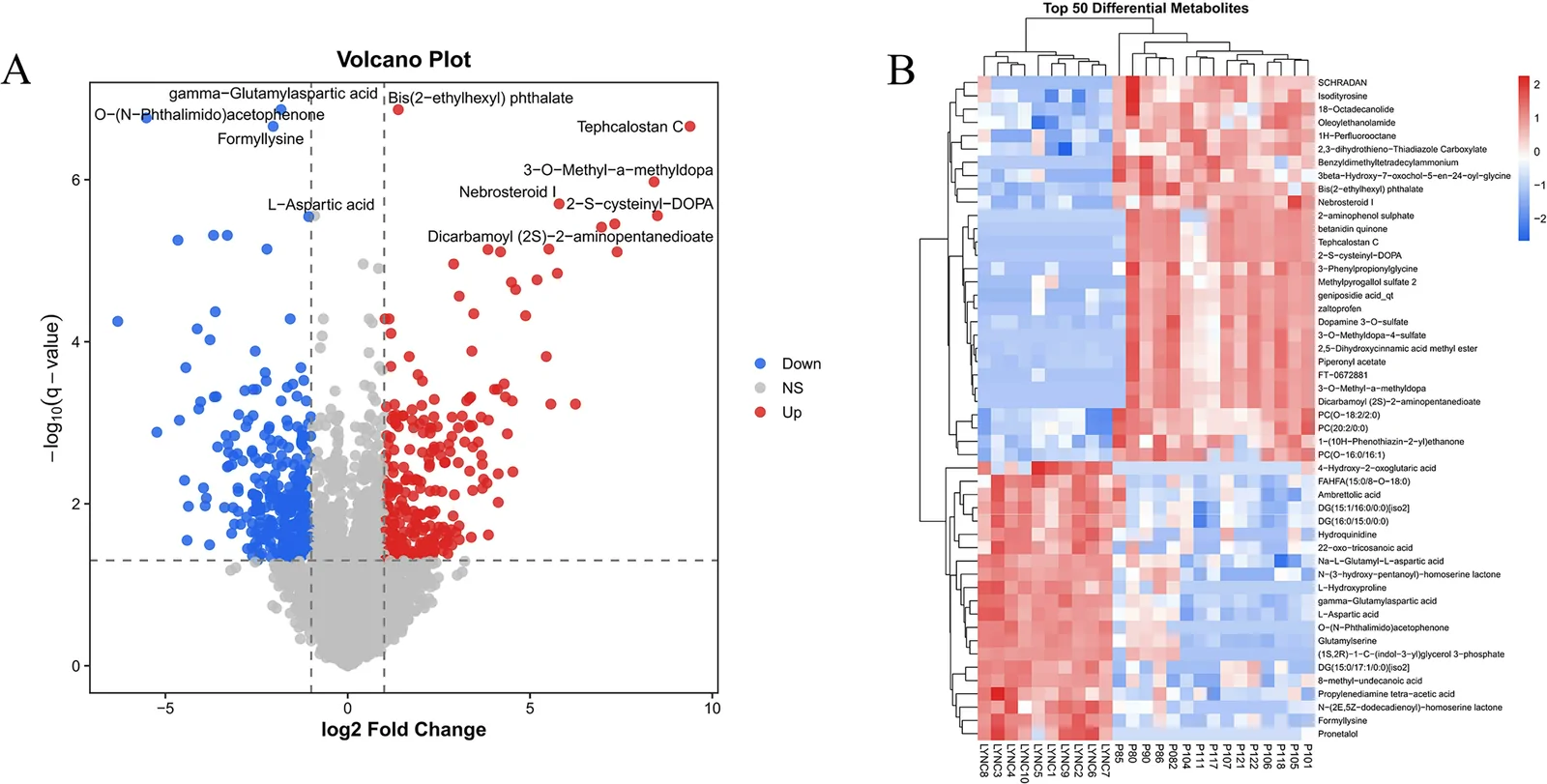
(A) Volcano plot: red dots represent upregulated metabolites (FC ≥ 2.0), blue dots represent downregulated metabolites (FC ≤ 0.5), and gray dots indicate non-significant changes. (B) Heatmap plot: the top 50 significantly differential metabolites ranked by *P*-value.

### Prioritization of candidate metabolites *via* machine learning

To further prioritize candidate metabolites from the 570 DEMs, two complementary machine learning approaches were applied. Both models were implemented with 10-fold cross-validation to reduce overfitting and improve model stability. First, Random Forest analysis was conducted to rank the importance of DEMs based on their contribution to group classification. Using 600 trees (ntree = 600), metabolites were ranked according to the mean decrease in Gini impurity, and the top five ranked features were selected (Fig. 5A). In parallel, LASSO regression was performed for feature selection *via* regularization, with the optimal penalty parameter (λ) determined through cross-validation. This approach identified 13 metabolites with non-zero coefficients (Figs. 5B, 5C). Among these candidates, 3-phenylpropionylglycine was consistently prioritized and suggested a potential trend for group discrimination in this exploratory context (Fig. 5D). However, given the pilot-scale nature of the metabolomics cohort (15 PD *vs*. 10 HCs) and the large number of metabolic features tested, these machine learning-derived features must be interpreted strictly as preliminary, hypothesis-generating signals. They were used solely to narrow down candidate metabolites for downstream genetic validation by Mendelian randomization, and should not be regarded as validated or robust diagnostic biomarkers at this stage.

**Figure 5:**
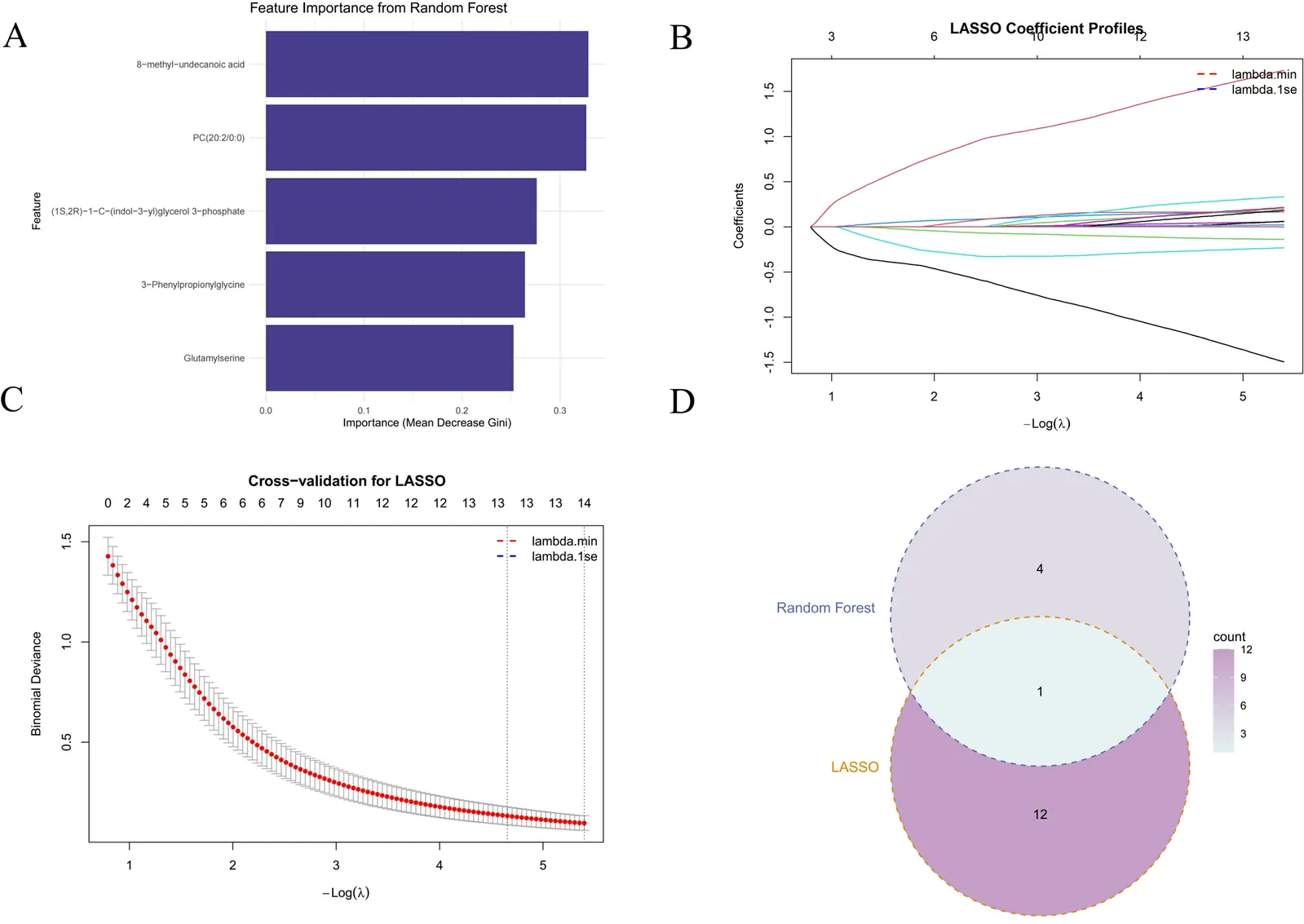
(A) Top five DEMs identified by the random forest model. (B) Coefficient trajectories of LASSO regression with changing λ values. (C) Ten-fold cross-validation for selecting the optimal λ in the LASSO regression model. (D) Venn diagram of the top five DEMs screened by random forest and LASSO regression models.

### Pathway enrichment analysis

KEGG pathway enrichment analysis was performed on the identified 570 DEMs. Among the top 19 significantly enriched pathways (Fig. 6A), metabolism-related pathways included caffeine metabolism, linoleic acid metabolism, sphingolipid metabolism, arachidonic acid metabolism, alpha-linolenic acid metabolism, histidine metabolism, and primary bile acid biosynthesis. Human disease-associated pathways encompassed choline metabolism in cancer, chemical carcinogenesis—receptor activation, prostate cancer, leishmaniasis, AGE-RAGE signaling in diabetic complications, and endocrine resistance. Organismal systems pathways included bile secretion, PPAR signaling, neurotrophin signaling, adipocytokine signaling, and GnRH secretion. Additionally, neuroactive ligand–receptor interaction (Environmental Information Processing) and necroptosis (Cellular Processes) pathways were enriched. These pathways collectively involve energy metabolism, amino acid and lipid metabolism, and neural signaling, indicating systemic metabolic dysregulation in PD. Notably, caffeine metabolism was the most significantly enriched pathway.

**Figure 6:**
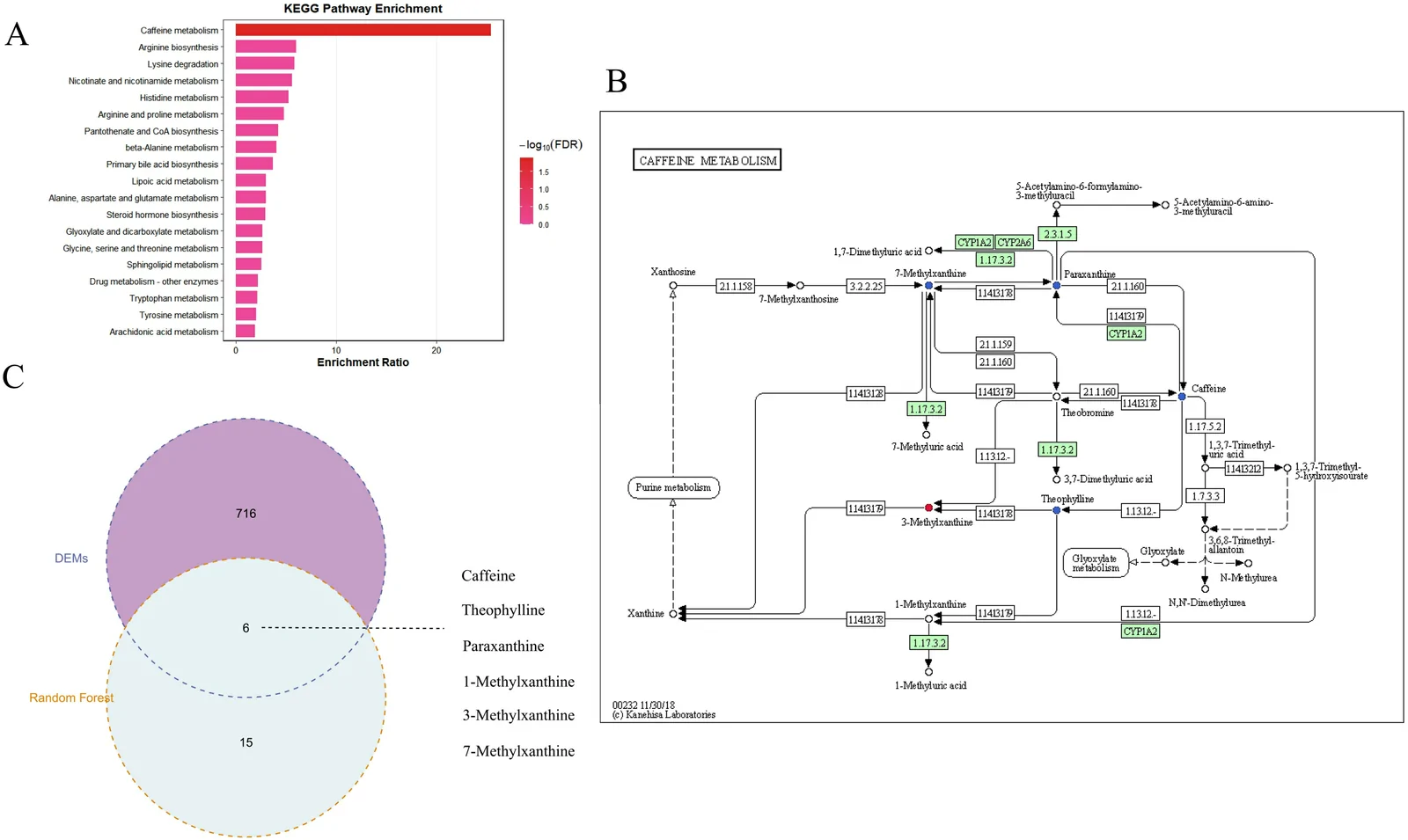
(A) KEGG pathway enrichment. Bar length indicates –log10 *P*-value; numbers at bar ends denote the count of differential metabolites. Bars are colored by KEGG Level 1 categories. (B) Caffeine metabolism in KEGG. Circles represent metabolites, colored by regulation in PD patients (red: upregulated; blue: downregulated). (C) Venn diagram showing the overlap between caffeine-related metabolites and DEMs.

To visualize the expression patterns of metabolites within this pathway, the KEGG Pathway Mapper was used, with metabolites color-coded according to their up or downregulation in PD (Fig. 6B), providing an intuitive reference for functional interpretation. In total, 21 differential metabolites were mapped to the caffeine metabolism pathway. Among them, six metabolites overlapped with the identified 570 DEMs, highlighting their potential importance. These six DEMs (caffeine, theophylline, paraxanthine, 1-methylxanthine, 3-methylxanthine, and 7-methylxanthine) were therefore considered key metabolites and were subsequently selected for MR analysis (Fig. 6C).

### MR analysis of exposures

Key DEMs identified through machine learning and KEGG pathway analyses were used as exposures in two-sample MR. SNP instruments for each exposure were selected as described in ‘Selection of Instrumental Variables for MR’, applying genome-wide significance thresholds, LD pruning (r^2^ < 0.001, 10,000 kb window), and instrument strength filtering (F > 10). For each included exposure, the number of SNP instruments, mean and median F-statistics, and the proportion of variance explained (R^2^) are summarized in Table 2. These metrics demonstrate that the selected instruments are sufficiently strong and independent, ensuring the robustness of the MR analyses.

**Table 2:** Instrumental variable characteristics for MR exposures.

Exposure (phenotype)	Population	N SNPs	Mean F-statistic	Mean R^2^	Sample size	Datasets in the GWAS	Year
3-Phenylpropionylglycine	European	13	23.77	0.06	405	GCST90319184	2024
Caffeine	European	4	29.56	0.07	415	GCST90319270	2024
Theophylline	European	10	23.10	0.01	2,602	GCST90317922	2024
Paraxanthine	European	15	23.60	0.01	2,311	GCST90317925	2024
1-Methylxanthine	European	13	22.08	0.02	1,016	GCST90319002	2024
3-Methylxanthine	European	13	23.18	0.01	2,602	GCST90317957	2024
7-Methylxanthine	European	12	22.83	0.01	2,602	GCST90317990	2024

**Note:**

CSF, cerebrospinal fluid; N SNPs, number of SNPs.

### DEMs randomization of metabolites and PD

Among the prioritized differential metabolites, five metabolites (caffeine, theophylline, paraxanthine, 1-methylxanthine, and 3-methylxanthine) did not show evidence of causal associations with PD risk (*P*_IVW_ > 0.05). Given the lack of statistical evidence for association, these metabolites were not subjected to further sensitivity analyses. In contrast, 3-phenylpropionylglycine was inversely associated with PD risk (OR = 0.8197, 95% CI [0.7248–0.9271], *P*_*FDR*_ = 0.0077), and 7-methylxanthine also showed a similar inverse association (OR = 0.8346, 95% CI [0.7226–0.9640], *P*_*FDR*_ = 0.0349) (Table 3, Fig. 7).

**Table 3:** MR analysis of the effect of DEMs on PD.

Exposure	Outcome	Method	*P*	FDR	OR (95%)	Cochran’s Q	Ph	Egger intercept	P_intercept_	P_global test_
Glutamylserine	PD	MR Egger	0.8524		0.9685 [0.6979–1.3440]	9.43	0.40	−0.01	0.81	0.52
		Weighted median	0.5288		0.9418 [0.7815–1.1350]					
		IVW	0.3294	0.8180	0.9332 [0.8120–1.0723]	9.50	0.49			
		Simple mode	0.6543		0.9446 [0.7413–1.2035]					
		Weighted mode	0.6444		0.9446 [0.7468–1.1947]					
3-Phenylpropionylglycine	PD	MR Egger	0.5037		0.8729 [0.5944–1.2818]	13.87	0.19	−0.01	0.73	0.26
		Weighted median	0.0265		0.8427 [0.7246–0.9802]					
		IVW	0.0015	0.0077	0.8197 [0.7248–0.9271]	14.03	0.23			
		Simple mode	0.4292		0.9018 [0.7046–1.1542]					
		Weighted mode	0.2113		0.8727 [0.7138–1.0671]					
Caffeine	PD	MR Egger	0.3772		1.1316 [0.9125–1.4034]	0.78	0.68	−0.4	0.34	0.66
		Weighted median	0.4834		1.0222 [0.9613–1.0870]		0.63			
		IVW	0.4274	0.6261	1.0209 [0.9700–1.0745]	1.71				
		Simple mode	0.6261		1.0235 [0.9408–1.1135]					
		Weighted mode	0.5869		1.0272 [0.9418–1.1204]					
1-Methylxanthine	PD	MR Egger	0.9588		1.0197 [0.4946–2.1024]	12.78	0.24	−0.00	0.90	0.31
		Weighted median	0.8914		0.9773 [0.7025–1.3595]					
		IVW	0.8548	0.9588	0.9764 [0.7560–1.2610]	12.80	0.31			
		Simple mode	0.8390		1.0621 [0.6022–1.8733]					
		Weighted mode	0.5752		1.1407 [0.7297–1.7830]					
3-Methylxanthine	PD	MR Egger	0.0330		0.5870 [0.3847–0.8955]	9.36	0.50	0.04	0.02	0.21
		Weighted median	0.3429		0.8758 [0.6657–1.1520]					
		IVW	0.3900	0.3929	0.9076 [0.7274–1.1322]	14.52	0.21			
		Simple mode	0.3929		0.8025 [0.4941–1.3034]					
		Weighted mode	0.2611		0.7984 [0.5501–1.1587]					
7-Methylxanthine	PD	MR Egger	0.0104		0.7368 [0.6091–0.8913]	9.29	0.50			
		Weighted median	0.1107		0.8552 [0.7057–1.0364]					
		IVW	0.0140	0.0349	0.8346 [0.7226–0.9640]	12.59	0.32	0.03	0.07	0.37
		Simple mode	0.8452		0.9660 [0.6883–1.3557]					
		Weighted mode	0.0668		0.8190 [0.6757–0.9928]					
Paraxanthine	PD	MR Egger	0.9827		0.9962 [0.7143–1.3895]	10.47	0.57	0.00	0.86	0.73
		Weighted median	0.4004		0.9093 [0.7286–1.1349]					
		IVW	0.7982	0.9827	1.0207 [0.8721–1.1948]	10.50	0.65			
		Simple mode	0.4951		0.8763 [0.6060–1.2672]					
		Weighted mode	0.3559		0.8594 [0.6302–1.1720]					
Theophylline	PD	MR Egger	0.6124		1.0974 [0.7767–1.5507]	4.70	0.79	−0.01	0.45	0.82
		Weighted median	0.7238		0.9582 [0.7562–1.2142]					
		IVW	0.8129	0.9816	0.9784 [0.8166–1.1723]	5.29	0.81			
		Simple mode	0.8450		1.0372 [0.7268–1.4802]					
		Weighted mode	0.9816		1.0043 [0.7062–1.4281]					

**Note:**

Odds ratios (ORs), 95% confidence intervals (95% CIs), and *P* values were calculated for the respective methods of MR analysis. The heterogeneity test in the IVW methods was performed *via* Cochran’s Q statistic and the global test for the MR-PRESSO method. *P* < 0.05 was considered significant. IVW, inverse-variance weighted; Ph, *P* value for heterogeneity test; P_intercept_, *P* value for the intercept of MR‒Egger regression. P global test, *P* value for the global test of MR-PRESSO.

**Figure 7:**
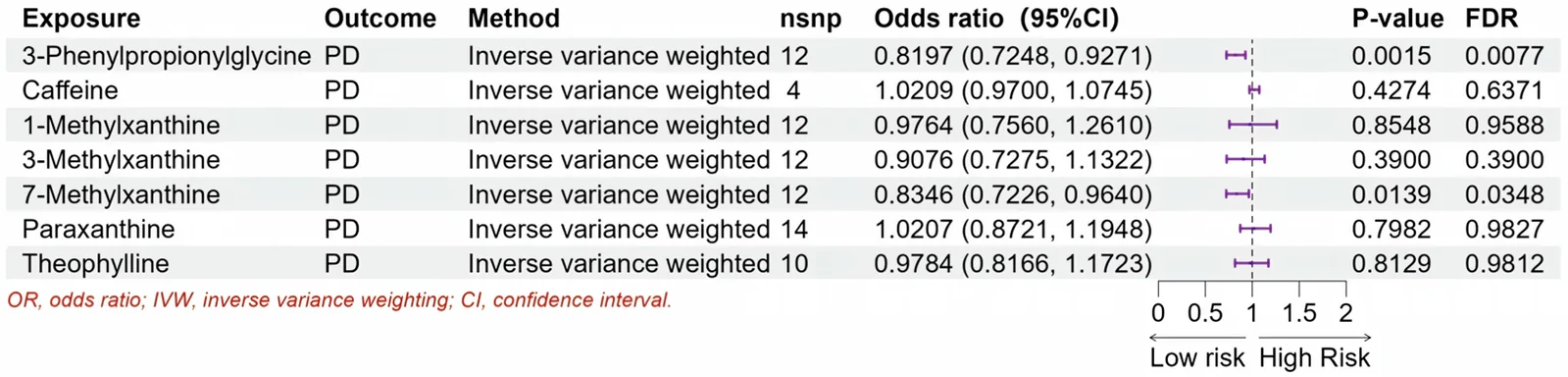
Forest plot of IVW MR analysis evaluating the putative causal association of metabolites on PD risk.

Sensitivity analyses supported the robustness of these findings. Cochran’s Q test indicated no evidence of heterogeneity for either 3-phenylpropionylglycine (*Q* = 14.03, df = 11, *P* = 0.23) or 7-methylxanthine (*Q* = 12.59, df = 11, *P* = 0.32). MR-Egger intercept tests did not detect evidence of horizontal pleiotropy (*P* > 0.05). MR-PRESSO analysis identified no outlier SNPs, indicating that the causal estimates were unlikely to be influenced by horizontal pleiotropy. Scatter plots and forest plots illustrated SNP-specific effects, while funnel plots showed no obvious asymmetry, and leave-one-out analyses further supported the robustness of the results (Figs. S1 and S2). These potential causal associations should be interpreted as exploratory and preliminary due to the small-scale discovery metabolomics cohort and cross-cohort integration design.

### MR analysis of circulating inflammatory proteins and PD

Based on the instrumental variable selection criteria described in ‘Selection of Instrumental Variables for MR’ and the instrument strength parameters summarized in Table S2, two-sample MR analyses identified three circulating inflammatory proteins—Flt3L, CCL13, and SULT1A1—that showed evidence of causal associations with PD risk after FDR correction. Genetically predicted higher levels of CCL13 were associated with an increased risk of PD (*P*_*FDR*_ = 0.0497, OR = 1.1077, 95% CI [1.0048–1.2210]), whereas higher levels of Flt3L (*P*_*FDR*_ = 0.0307; OR = 0.8948, 95% CI [0.8264–0.9688]) and SULT1A1 (*P*_*FDR*_ = 0.0133; OR = 0.8720, 95% CI [0.7989–0.9535]) were associated with a reduced risk of PD.

Sensitivity analyses indicated no strong evidence of horizontal pleiotropy or heterogeneity. The MR-Egger intercept tests were not statistically significant (*P* > 0.05), and MR-PRESSO global tests did not identify significant outliers. Estimates derived from MR-Egger and weighted median methods were directionally consistent with the primary IVW results. Visualization analyses, including scatter plots, forest plots, and funnel plots, did not indicate substantial heterogeneity or asymmetry. Leave-one-out analyses further supported the stability of the findings (Table 4; Figs. 8, S3–S6).

**Table 4:** MR analysis of the effect of inflammatory proteins on PD.

Exposure	Outcome	Method	*P*-value	FDR	OR (95%)	Cochran’s Q	Ph	Egger intercept	P_intercept_	P_globaltest_
FIt3L	PD	MR Egger	0.2559		0.9227 [0.8046–1.0581]	34.68	0.81	−0.00	0.59	0.83
		Weighted median	0.4130		0.9473 [0.8322–1.0783]					
		IVW	0.0061	0.0307	0.8948 [0.8264–0.9688]	34.97	0.83			
		Simple mode	0.5167		0.9244 [0.7304–1.1700]					
		Weighted mode	0.3693		0.9424 [0.8291–1.0713]					
SULT1A1	PD	MR Egger	0.1813		0.8602 [0.6930–1.0676]	39.42	0.17	0.00	0.89	0.14
		Weighted median	0.1054		0.8932 [0.7791–1.0240]					
		IVW	0.0027	0.0133	0.8720 [0.7975–0.9535]	39.44	0.20			
		Simple mode	0.3499		0.8883 [0.6953–1.1347]					
		Weighted mode	0.2584		0.8984 [0.7484–1.0784]					
CCL13	PD	MR Egger	0.0132		1.2869 [1.0691–1.5491]	30.71	0.20	−0.02	0.08	0.14
		Weighted median	0.0023		1.2102 [1.0708–1.3677]					
		IVW	0.0398	0.0497	1.1077 [1.0048–1.2210]	34.85	0.11			
		Simple mode	0.4111		1.0926 [0.8876–1.3449]					
		Weighted mode	0.0102		1.1852 [1.0510–1.3366]					

**Note:**

Odds ratios (ORs), 95% confidence intervals (95% CIs), and *P* values were calculated for the respective methods of MR analysis. The heterogeneity test in the IVW methods was performed *via* Cochran’s Q statistic and the global test for the MR-PRESSO method. *P* < 0.05 was considered significant. IVW, inverse-variance weighted; Ph, *P* value for heterogeneity test; P_intercept_, *P* value for the intercept of MR‒Egger regression. P global test, *P* value for the global test of MR-PRESSO.

**Figure 8:**
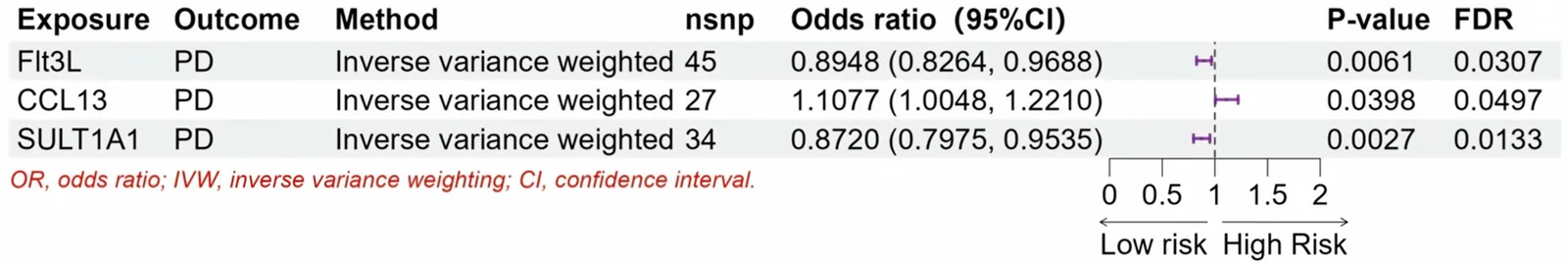
Forest plot of IVW MR analysis evaluating the causal effects of inflammatory proteins on PD risk.

### MR for mediation analysis

To explore whether circulating inflammatory proteins mediated the effects of prioritized metabolites on PD, a two-step MR mediation framework was applied. This approach first assessed the putative causal association of metabolites on inflammatory proteins, followed by evaluation of the effects of these proteins on PD risk.

Among the tested metabolite–protein–PD pathways, a potential mediation effect was identified for the axis involving 3-phenylpropionylglycine and SULT1A1 (Table 5). Specifically, genetically predicted higher levels of 3-phenylpropionylglycine were associated with decreased SULT1A1 levels (β = −0.07, *P* = 0.01), while higher SULT1A1 levels were associated with reduced PD risk (β = −0.14, *P* = 0.01). Consistent with these two-step associations, the total effect of 3-phenylpropionylglycine on PD was negative (β = −0.19), whereas the estimated indirect effect was positive (β = 0.01), yielding a mediation proportion of 5.15%. This directionality is mathematically consistent with the product-of-coefficients mediation framework, as both the exposure–mediator and mediator–outcome effects were negative. Notably, the estimated mediation proportion of 5.15% indicates only a modest indirect effect, suggesting that SULT1A1 explains only a small proportion of the association between 3-phenylpropionylglycine and PD and should not be interpreted as the primary mediator of this relationship.

**Table 5:** Mediation MR results.

Exposure	Mediator	β (X → M)	*P* (X → M)	β (M → Y)	*P* (M → Y)_st_	Total effect β	Indirect effect β	Proportion mediated (%)
3-Phenylpropionylglycine	SULT1A1	−0.07	0.01	−0.14	0.01	−0.19	0.01	5.15

**Note:**

X, exposure; M, mediator; Y, outcome.

## Discussion

This study implemented an exploratory integrative framework combining pilot-scale clinical metabolomics with a two-sample MR analysis to systematically investigate potential links between circulating metabolites, inflammatory proteins, and PD risk. By leveraging machine learning-based feature prioritization and genetic instruments, our findings provide preliminary, hypothesis-generating evidence supporting a potential metabolic-immune axis in PD, strengthening causal inference compared with conventional observational studies. Given the large number of tested metabolomic features relative to our restricted discovery sample size (15 PD cases *vs*. 10 HCs), all identified candidates are framed strictly as preliminary leads rather than robust or clinically applicable biomarkers, requiring extensive validation in larger independent cohorts.

3-Phenylpropionylglycine (3-PPG) is an aromatic metabolite derived from gut microbiota and serves as an important marker of host–microbial metabolic interactions (Jariyasopit & Khoomrung, 2023). Our MR analysis revealed a significant inverse causal association between 3-PPG and PD risk, suggesting its potential neuroprotective role. Biochemically, 3-PPG is generated in the mitochondrial matrix from cinnamoyl-CoA and glycine *via* glycine N-acyltransferase (GLYAT). This reaction represents a classic glycine conjugation detoxification pathway that facilitates the clearance of aromatic acyl-CoAs (Badenhorst et al., 2013; van der Sluis et al., 2017). Previous studies have shown that mitochondrial metabolic dysregulation and oxidative stress play key roles in PD pathogenesis, highlighting the importance of maintaining mitochondrial metabolic homeostasis for dopaminergic neuron protection (Marzetti et al., 2026; Tran et al., 2026). In this context, the observed association may reflect a broader link between glycine conjugation-related metabolic efficiency and neuronal resilience. However, the exact biological role of 3-PPG in the central nervous system remains uncertain, and direct mechanistic evidence is currently limited.

Mediation MR analysis further suggested that SULT1A1 may be involved in the association between 3-PPG and PD risk. SULT1A1 is a sulfotransferase enzyme implicated in catecholamine metabolism and detoxification processes, and may contribute to the regulation of dopamine-derived oxidative metabolites (Salman, Kadlubar & Falany, 2009; Sidharthan, Minchin & Butcher, 2013). In our analysis, 3-PPG was inversely associated with SULT1A1 levels, while SULT1A1 was also inversely associated with PD risk, yielding an estimated mediation proportion of approximately 5.15%. This modest indirect effect suggests that SULT1A1 is likely one contributor among multiple biological pathways rather than the principal mediator linking 3-phenylpropionylglycine with PD. Given the small magnitude of mediation and the strong assumptions required for causal mediation MR—including the absence of unmeasured confounding and no horizontal pleiotropy in the mediator–outcome pathway—these findings should therefore be interpreted cautiously and regarded as hypothesis-generating pending independent validation.

Our pathway enrichment analysis strongly highlighted caffeine metabolism as a significantly altered pathway in PD. Previous studies have shown that long-term caffeine intake can downregulate dopamine transporters and reduce PD risk (Saarinen et al., 2024), and cohort studies indicate that habitual unsweetened coffee consumption further lowers PD incidence (Zhao et al., 2024; Zhang et al., 2024). Metabolomic analysis revealed enrichment of PD-related differential metabolites within the caffeine metabolism pathway, prompting us to perform MR on overlapping caffeine-derived metabolites. The analysis indicated that 7-methylxanthine exerts a causal inverse association against PD.

Evidence suggests that caffeine and its downstream metabolites possess antioxidant and anti-inflammatory properties, reducing intracellular reactive oxygen species (ROS) accumulation, alleviating oxidative stress-induced damage to dopaminergic neurons, and suppressing PD-related inflammatory signaling and microglial activation (Geue et al., 2025; Epplen et al., 2025). Although direct mechanistic studies on 7-methylxanthine remain limited, its role as an adenosine receptor antagonist suggests it may mediate similar antioxidant and anti-inflammatory effects, which underlie caffeine’s potentially neuroprotective actions in PD models (Ikram et al., 2020). Furthermore, caffeine has been reported to inhibit NLRP3 inflammasome activation and mitigate inflammation by reducing ROS generation and downregulating pro-inflammatory pathways, implying that xanthine metabolites may attenuate α-synuclein–induced neuroinflammation (Vargas-Pozada et al., 2022). Oxidative stress and lipid peroxidation are also key triggers of ferroptosis; thus, 7-methylxanthine may indirectly reduce the initiation of ferroptotic cell death by modulating intracellular ROS levels and enhancing antioxidant defenses. Xanthine derivatives may additionally improve neuronal survival by regulating energy metabolism and adenosine receptor signaling, further potentiating their potentially neuroprotective effects (Kasabova-Angelova et al., 2020). However, direct functional evidence for 7-methylxanthine in the central nervous system remains limited, and it should be interpreted as a supportive, hypothesis-generating metabolic signal rather than definitive proof of protection.

A critical methodological integration in this study involves using CSF metabolite GWAS summary statistics as instruments for our plasma-derived metabolomic findings. We specifically selected CSF-derived genetic instruments because they directly reflect central nervous system metabolism, which holds greater pathophysiological relevance to the neurodegenerative processes of PD than peripheral blood. CSF is in direct, contiguous contact with the brain parenchyma, thereby serving as a superior proxy for capturing metabolic alterations in dopaminergic pathways and local neuroinflammatory microenvironments. Nevertheless, this cross-compartment alignment introduces an inherent limitation and biological mismatch. Interpreting results across different biological compartments can lead to divergence, as metabolite transport, genetic determinants, and concentration ranges are strictly regulated by the blood-brain and blood-CSF barriers. Consequently, a genetic variant influencing central CSF metabolite traits may not invariably parallel concentration changes captured in peripheral plasma, adding a layer of uncertainty to the integrated MR framework that necessitates highly transparent and cautious interpretation.

Furthermore, potential confounding from unmeasured clinical and lifestyle variables requires detailed discussion. A key limitation of our pilot-scale discovery cohort is the lack of granular, standardized records regarding patients’ current dopaminergic or other anti-parkinsonian medication status at the time of blood sampling. Because dopaminergic therapies can profoundly alter systemic and central metabolomic profiles, this lack of data introduces residual confounding that could not be adjusted for during the discovery stage. Similarly, detailed clinical phenotypes including disease duration, disease severity, and lifestyle variables such as dietary habits and habitual caffeine or tea intake were unavailable. Given our prominent findings involving caffeine-related pathways, we cannot completely rule out the possibility that the differential plasma expression of these metabolites was partially driven by lifestyle variations or alterations in caffeine consumption secondary to prodromal symptoms. While the two-sample MR framework substantially mitigates reverse causation by utilizing randomly allocated genetic variants as lifelong proxies, future clinical validation studies must rigorously collect and adjust for dietary records, precise medication status, and clinical severity scores to fully isolate true pathophysiological alterations.

However, several limitations of this study should be acknowledged. First, metabolomic data in the discovery cohort were obtained from plasma samples. However, due to limitations in publicly available resources, the corresponding genetic instruments for metabolites were derived from publicly available GWAS datasets of CSF metabolites. Depending on the original study design, such datasets may reflect metabolite levels in different biological compartments, including circulating plasma and CSF. Therefore, potential discrepancies between plasma-derived metabolomic profiles and genetically predicted metabolite levels across distinct biological compartments may introduce uncertainty in result interpretation. Although preliminary analyses did not indicate significant associations for excluded metabolites, differences in genetic architecture and metabolic profiles across populations warrant cautious interpretation of findings in individuals of European ancestry. Second, while the GWAS sample size for PD was sufficient to ensure robust association analyses, some GWAS datasets for immune-related factors were relatively small, which may limit statistical power and increase uncertainty in causal effect estimates. Third, although strict instrumental variable selection criteria were applied to minimize confounding bias, horizontal pleiotropy cannot be completely excluded in Mendelian randomization analyses. Therefore, observed associations should be interpreted with caution and validated in independent datasets and experimental studies. Fourth, in selecting SNPs strongly associated with metabolites and inflammatory factors, thresholds of 1 × 10^−6^ and 1 × 10^−5^ were applied. This approach aimed to balance the discovery of true associations and control false positives, but some risk of spurious findings remains, as no statistical threshold can completely eliminate error. Finally, although Mendelian randomization provides a robust framework for causal inference, it relies on key assumptions, including linearity and absence of effect modification, which may not fully capture biological complexity and individual heterogeneity. Therefore, MR findings should be interpreted as supportive evidence for potential causal mechanisms rather than definitive proof of clinical causality. Randomized controlled trials and functional experimental studies remain essential for validation of these results.

Despite the preliminary and exploratory nature of our findings, this integrative pilot study outlines a potential roadmap for future translational research in PD. First, the identified hypothesis-generating signals, specifically 3-phenylpropionylglycine and 7-methylxanthine, provide narrow, high-priority targets for future targeted metabolomics validation studies utilizing absolute quantification in large-scale, multi-center cohorts. Mechanistically, if these preliminary protective signals are validated through *in vitro* and *in vivo* functional experiments, they could assist in therapeutic pathway identification, potentially opening new avenues for metabolism- or inflammation-based intervention strategies (such as gut microbiota modulation for 3-PPG or tailored xanthine derivative treatments). Moreover, integrating genetically predicted metabolic risk scores with clinical phenotypes might eventually contribute to personalized risk stratification and early biomarker development in prodromal PD. However, we emphasize that these translational applications remain speculative at this stage, and any clinical translation remains strictly contingent upon rigorous experimental validation and extensive prospective clinical evaluation.

## Conclusion

By integrating pilot-scale plasma metabolomics with MR, this exploratory study identifies 3-PPG and 7-methylxanthine as preliminary candidate signals inversely associated with PD risk, with SULT1A1 emerging as a potential downstream inflammatory mediator. However, given the small discovery sample size, the cross-compartment alignment (plasma *vs*. CSF), and potential confounding from medication or dietary habits, these findings must be interpreted with caution. They should be viewed strictly as preliminary, hypothesis-generating leads rather than robust biomarkers. Ultimately, large-scale longitudinal cohorts and functional experiments are fundamentally required to validate these metabolic candidates and evaluate their future translational relevance.

## Supplemental Information

10.7717/peerj.21670/supp-1 Supplemental Information 1 Supplemental tables and figures.

10.7717/peerj.21670/supp-2 Supplemental Information 2 Raw data.

## Abbreviations

PD: Parkinson’s disease
LC-MS: Liquid Chromatography-Mass Spectrometry
DEMs: Differentially expressed metabolites
KEGG: Kyoto Encyclopedia of Genes and Genomes
MR: Mendelian randomization
SNPs: Single nucleotide polymorphisms
IVs: Instrumental variables
MDS: Movement Disorder Society
BMI: Body mass index
LASSO: Least absolute shrinkage and selection operator
RF: Random Forest
HCs: Healthy controls
CSF: Cerebrospinal fluid
ESI: Electrospray ionization
PCA: Principal Component Analysis

## Additional Information and Declarations

### Data Availability

The following information was supplied regarding data availability:

The data is available in the Supplemental Files.

The metabolomic data is available at Metabolights: https://www.ebi.ac.uk/metabolights/editor/MTBLS13269/overview.
