# Supplementary material for: Exploring metabolic candidates and inflammatory mediation in Parkinson’s disease: a pilot integrative metabolomics and Mendelian randomization study
Source: PeerJ. 2026 Sep 28;14:e21670. doi: 10.7717/peerj.21670 (PMC13629568; doi:10.7717/peerj.21670)
Supplement: Supplementary file 1 [file peerj-14-21670-s001.docx]

**Supplementary materials**

Table 1. Baseline characteristics of data sources

| Year | Phenotype | Population | Sample size | Datasets ID | pQTL classification |  |
| --- | --- | --- | --- | --- | --- | --- |
| Exposure |  |  |  |  |  |  |
| Differential metabolite |  |  |  |  |  |  |
| 2024 | 3-phenylpropionylglycine | European | 405 | GCST90319184 | - |  |
| 2024 | Caffeine | European | 415 | GCST90319270 | - |  |
| 2024 | Theophylline | European | 2602 | GCST90317922 | - |  |
| 2024 | Paraxanthine | European | 2311 | GCST90317925 | - |  |
| 2024 | 1-Methylxanthine | European | 1016 | GCST90319002 | - |  |
| 2024 | 3-Methylxanthine | European | 2602 | GCST90317957 | - |  |
| 2024 | 7-Methylxanthine | European | 2602 | GCST90317990 | - |  |
| Mediators  (Inflammatory proteins) |  |  |  |  |  |  |
| 2023 | ADA | European | 14736 | GCST90274759 | Cis + trans |  |
| 2023 | ARTN | European | 11778 | GCST90274760 | Cis + trans |  |
| 2023 | AXIN1 | European | 11793 | GCST90274761 | Cis + trans |  |
| 2023 | Beta-NGF | European | 14743 | GCST90274762 | Cis + trans |  |
| 2023 | CASP-8 | European | 14744 | GCST90274763 | Cis + trans |  |
| 2023 | CCL19 | European | 14736 | GCST90274765 | Cis + trans |  |
| 2023 | CCL20 | European | 14736 | GCST90274766 | Cis + trans |  |
| 2023 | CCL23 | European | 14736 | GCST90274767 | Cis + trans |  |
| 2023 | CCL25 | European | 14736 | GCST90274768 | Cis + trans |  |
| 2023 | CCL28 | European | 14734 | GCST90274769 | Cis + trans |  |
| 2023 | CCL4 | European | 14744 | GCST90274770 | Cis + trans |  |
| 2023 | CD40 | European | 14736 | GCST90274772 | Cis + trans |  |
| 2023 | CDCP1 | European | 14734 | GCST90274775 | Cis + trans |  |
| 2023 | CXCL1 | European | 14736 | GCST90274779 | Cis + trans |  |
| 2023 | CXCL10 | European | 14744 | GCST90274780 | Cis + trans |  |
| 2023 | CXCL11 | European | 14736 | GCST90274781 | Cis + trans |  |
| 2023 | CXCL5 | European | 14736 | GCST90274782 | Cis + trans |  |
| 2023 | CXCL6 | European | 14744 | GCST90274783 | Cis + trans |  |
| 2023 | CXCL9 | European | 14735 | GCST90274784 | Cis + trans |  |
| 2023 | CST5 | European | 14736 | GCST90274777 | Cis + trans |  |
| 2023 | DNER | European | 14735 | GCST90274785 | Cis + trans |  |
| 2023 | CCL11 | European | 14734 | GCST90274764 | Cis + trans |  |
| 2023 | EIF4EBP1 | European | 14736 | GCST90274758 | Cis + trans |  |
| 2023 | FGF-19 | European | 14744 | GCST90274787 | Cis + trans |  |
| 2023 | FGF-21 | European | 14743 | GCST90274788 | Cis + trans |  |
| 2023 | FGF-23 | European | 14735 | GCST90274789 | Cis + trans |  |
| 2023 | FGF-5 | European | 11789 | GCST90274790 | Cis + trans |  |
| 2023 | FIt3L | European | 14734 | GCST90274791 | Cis + trans |  |
| 2023 | CX3CL1 | European | 14743 | GCST90274778 | Cis + trans |  |
| 2023 | hGDNF | European | 14736 | GCST90274792 | Cis + trans |  |
| 2023 | HGF | European | 14734 | GCST90274793 | Cis + trans |  |
| 2023 | IFN-gamma | European | 11793 | GCST90274794 | Cis + trans |  |
| 2023 | IL-1 alpha | European | 14744 | GCST90274795 | Cis + trans |  |
| 2023 | IL-10 | European | 11793 | GCST90274796 | Cis + trans |  |
| 2023 | IL-10RA | European | 14734 | GCST90274797 | Cis + trans |  |
| 2023 | IL10RB | European | 14735 | GCST90274798 | Cis + trans |  |
| 2023 | IL-12B | European | 11792 | GCST90274799 | Cis + trans |  |
| 2023 | IL-15RA | European | 11792 | GCST90274800 | Cis + trans |  |
| 2023 | IL-17A | European | 11784 | GCST90274801 | Cis + trans |  |
| 2023 | IL-17C | European | 11793 | GCST90274802 | Cis + trans |  |
| 2023 | IL-18 | European | 14744 | GCST90274803 | Cis + trans |  |
| 2023 | IL-18R1 | European | 14743 | GCST90274804 | Cis + trans |  |
| 2023 | IL-1α | European | 11788 | GCST90274805 | Cis + trans |  |
| 2023 | IL-2 | European | 11789 | GCST90274806 | Cis + trans |  |
| 2023 | IL-2RB | European | 11792 | GCST90274811 | Cis + trans |  |
| 2023 | IL-20 | European | 11784 | GCST90274807 | Cis + trans |  |
| 2023 | IL-20RA | European | 11792 | GCST90274808 | Cis + trans |  |
| 2023 | IL-22RA1 | European | 11793 | GCST90274809 | Cis + trans |  |
| 2023 | IL-24 | European | 11785 | GCST90274810 | Cis + trans |  |
| 2023 | IL-33 | European | 11793 | GCST90274812 | Cis + trans |  |
| 2023 | IL-4 | European | 11793 | GCST90274813 | Cis + trans |  |
| 2023 | IL-5 | European | 11792 | GCST90274814 | Cis + trans |  |
| 2023 | IL-6 | European | 14743 | GCST90274815 | Cis + trans |  |
| 2023 | IL-7 | European | 14736 | GCST90274816 | Cis + trans |  |
| 2023 | IL-8 | European | 14744 | GCST90274817 | Cis + trans |  |
| 2023 | LAP TGF-beta-1 | European | 14736 | GCST90274818 | Cis + trans |  |
| 2023 | LIF | European | 11793 | GCST90274819 | Cis + trans |  |
| 2023 | LIF-R | European | 11784 | GCST90274820 | Cis + trans |  |
| 2023 | CSF-1 | European | 14734 | GCST90274776 | Cis + trans |  |
| 2023 | MIP-1 alpha | European | 14743 | GCST90274825 | Cis + trans |  |
| 2023 | MMP-1 | European | 14744 | GCST90274826 | Cis + trans |  |
| 2023 | MMP-10 | European | 14744 | GCST90274827 | Cis + trans |  |
| 2023 | CCL2 | European | 14736 | GCST90274822 | Cis + trans |  |
| 2023 | CCL8 | European | 14733 | GCST90274821 | Cis + trans |  |
| 2023 | CCL7 | European | 11783 | GCST90274823 | Cis + trans |  |
| 2023 | CCL13 | European | 14736 | GCST90274824 | Cis + trans |  |
| 2023 | CD244 | European | 14735 | GCST90274771 | Cis + trans |  |
| 2023 | NT-3 | European | 14744 | GCST90274829 | Cis + trans |  |
| 2023 | NRTN | European | 11791 | GCST90274828 | Cis + trans |  |
| 2023 | OSM | European | 14736 | GCST90274831 | Cis + trans |  |
| 2023 | OPG | European | 14733 | GCST90274830 | Cis + trans |  |
| 2023 | PD-L1 | European | 14736 | GCST90274832 | Cis + trans |  |
| 2023 | EN-RAGE | European | 14743 | GCST90274786 | Cis + trans |  |
| 2023 | SLAMF1 | European | 14734 | GCST90274835 | Cis + trans |  |
| 2023 | SIRT2 | European | 14736 | GCST90274834 | Cis + trans |  |
| 2023 | STAMPB | European | 14736 | GCST90274837 | Cis + trans |  |
| 2023 | SCF | European | 14736 | GCST90274833 | Cis + trans |  |
| 2023 | SULT1A1 | European | 11793 | GCST90274836 | Cis + trans |  |
| 2023 | CD5 | European | 14735 | GCST90274773 | Cis + trans |  |
| 2023 | CD6 | European | 14735 | GCST90274774 | Cis + trans |  |
| 2023 | TSLP | European | 11793 | GCST90274845 | Cis + trans |  |
| 2023 | TNFB | European | 11792 | GCST90274840 | Cis + trans |  |
| 2023 | TRANCE | European | 14736 | GCST90274844 | Cis + trans |  |
| 2023 | TRAIL | European | 14735 | GCST90274843 | Cis + trans |  |
| 2023 | TGF-alpha | European | 14733 | GCST90274838 | Cis + trans |  |
| 2023 | TWEAK | European | 11785 | GCST90274839 | Cis + trans |  |
| 2023 | TNF | European | 14736 | GCST90274846 | Cis + trans |  |
| 2023 | TNFSF14 | European | 11793 | GCST90274842 | Cis + trans |  |
| 2023 | TNFRSF9 | European | 11784 | GCST90274841 | Cis + trans |  |
| 2023 | uPA | European | 14734 | GCST90274847 | Cis + trans |  |
| 2023 | VEGF_A | European | 14744 | GCST90274848 | Cis + trans |  |
| Outcome |  |  |  |  |  |  |
| 2024 | Parkinson's disease | European | 500348 | finngen_R12_G6_PARKINSON | - |  |

Table 2. Characteristics of instrumental variables for MR mediators

| Year | Phenotype | Population | Sample size | Datasets ID | N SNPs | Mean F-statistic | Mean R^2^ |
| --- | --- | --- | --- | --- | --- | --- | --- |
| 2023 | ADA | European | 14736 | GCST90274759 | 2166 | 17.45 | 0.002 |
| 2023 | ARTN | European | 11778 | GCST90274760 | 34 | 21.58 | 0.001 |
| 2023 | AXIN1 | European | 11793 | GCST90274761 | 13 | 22.11 | 0.001 |
| 2023 | Beta-NGF | European | 14743 | GCST90274762 | 39 | 22.99 | 0.001 |
| 2023 | CASP-8 | European | 14744 | GCST90274763 | 24 | 26.47 | 0.001 |
| 2023 | CCL19 | European | 14736 | GCST90274765 | 42 | 35.08 | 0.001 |
| 2023 | CCL20 | European | 14736 | GCST90274766 | 34 | 24.17 | 0.002 |
| 2023 | CCL23 | European | 14736 | GCST90274767 | 35 | 53.90 | 0.004 |
| 2023 | CCL25 | European | 14736 | GCST90274768 | 2179 | 18.50 | 0.001 |
| 2023 | CCL28 | European | 14734 | GCST90274769 | 47 | 23.18 | 0.002 |
| 2023 | CCL4 | European | 14744 | GCST90274770 | 36 | 66.16 | 0.005 |
| 2023 | CD40 | European | 14736 | GCST90274772 | 26 | 73.80 | 0.005 |
| 2023 | CDCP1 | European | 14734 | GCST90274775 | 38 | 39.78 | 0.003 |
| 2023 | CXCL1 | European | 14736 | GCST90274779 | 30 | 39.33 | 0.003 |
| 2023 | CXCL10 | European | 14744 | GCST90274780 | 36 | 29.94 | 0.002 |
| 2023 | CXCL11 | European | 14736 | GCST90274781 | 42 | 29.30 | 0.002 |
| 2023 | CXCL5 | European | 14736 | GCST90274782 | 27 | 61.30 | 0.004 |
| 2023 | CXCL6 | European | 14744 | GCST90274783 | 2176 | 17.18 | 0.005 |
| 2023 | CXCL9 | European | 14735 | GCST90274784 | 43 | 25.69 | 0.002 |
| 2023 | CST5 | European | 14736 | GCST90274777 | 2160 | 17.70 | 0.001 |
| 2023 | DNER | European | 14735 | GCST90274785 | 29 | 35.80 | 0.003 |
| 2023 | CCL11 | European | 14734 | GCST90274764 | 28 | 34.73 | 0.002 |
| 2023 | EIF4EBP1 | European | 14736 | GCST90274758 | 21 | 27.63 | 0.002 |
| 2023 | FGF-19 | European | 14744 | GCST90274787 | 36 | 28.14 | 0.002 |
| 2023 | FGF-21 | European | 14743 | GCST90274788 | 28 | 32.69 | 0.002 |
| 2023 | FGF-23 | European | 14735 | GCST90274789 | 30 | 24.70 | 0.002 |
| 2023 | FGF-5 | European | 11789 | GCST90274790 | 2132 | 17.39 | 0.001 |
| 2023 | FIt3L | European | 14734 | GCST90274791 | 51 | 34.46 | 0.002 |
| 2023 | CX3CL1 | European | 14743 | GCST90274778 | 35 | 32.45 | 0.002 |
| 2023 | hGDNF | European | 14736 | GCST90274792 | 28 | 38.37 | 0.003 |
| 2023 | HGF | European | 14734 | GCST90274793 | 35 | 24.79 | 0.002 |
| 2023 | IFN-gamma | European | 11793 | GCST90274794 | 21 | 22.21 | 0.002 |
| 2023 | IL-1 alpha | European | 14744 | GCST90274795 | 38 | 26.52 | 0.002 |
| 2023 | IL-10 | European | 11793 | GCST90274796 | 22 | 21.37 | 0.002 |
| 2023 | IL-10RA | European | 14734 | GCST90274797 | 30 | 70.70 | 0.004 |
| 2023 | IL10RB | European | 14735 | GCST90274798 | 2159 | 17.85 | 0.001 |
| 2023 | IL-12B | European | 11792 | GCST90274799 | 33 | 21.46 | 0.002 |
| 2023 | IL-15RA | European | 11792 | GCST90274800 | 31 | 54.66 | 0.005 |
| 2023 | IL-17A | European | 11784 | GCST90274801 | 27 | 21.57 | 0.002 |
| 2023 | IL-17C | European | 11793 | GCST90274802 | 37 | 22.76 | 0.002 |
| 2023 | IL-18 | European | 14744 | GCST90274803 | 34 | 34.86 | 0.002 |
| 2023 | IL-18R1 | European | 14743 | GCST90274804 | 2200 | 17.63 | 0.001 |
| 2023 | IL-1α | European | 11788 | GCST90274805 | 26 | 27.15 | 0.003 |
| 2023 | IL-2 | European | 11789 | GCST90274806 | 26 | 22.02 | 0.002 |
| 2023 | IL-2RB | European | 11792 | GCST90274811 | 26 | 21.48 | 0.002 |
| 2023 | IL-20 | European | 11784 | GCST90274807 | 26 | 21.38 | 0.002 |
| 2023 | IL-20RA | European | 11792 | GCST90274808 | 23 | 21.41 | 0.002 |
| 2023 | IL-22RA1 | European | 11793 | GCST90274809 | 24 | 21.32 | 0.002 |
| 2023 | IL-24 | European | 11785 | GCST90274810 | 24 | 21.40 | 0.002 |
| 2023 | IL-33 | European | 11793 | GCST90274812 | 29 | 20.97 | 0.002 |
| 2023 | IL-4 | European | 11793 | GCST90274813 | 26 | 21.41 | 0.002 |
| 2023 | IL-5 | European | 11792 | GCST90274814 | 26 | 21.56 | 0.002 |
| 2023 | IL-6 | European | 14743 | GCST90274815 | 15 | 33.91 | 0.002 |
| 2023 | IL-7 | European | 14736 | GCST90274816 | 27 | 23.22 | 0.002 |
| 2023 | IL-8 | European | 14744 | GCST90274817 | 34 | 23.45 | 0.002 |
| 2023 | LAP TGF-beta-1 | European | 14736 | GCST90274818 | 37 | 27.15 | 0.002 |
| 2023 | LIF | European | 11793 | GCST90274819 | 32 | 20.99 | 0.002 |
| 2023 | LIF-R | European | 11784 | GCST90274820 | 32 | 32.90 | 0.003 |
| 2023 | CSF-1 | European | 14734 | GCST90274776 | 32 | 27.02 | 0.002 |
| 2023 | MIP-1 alpha | European | 14743 | GCST90274825 | 23 | 67.30 | 0.005 |
| 2023 | MMP-1 | European | 14744 | GCST90274826 | 31 | 57.95 | 0.004 |
| 2023 | MMP-10 | European | 14744 | GCST90274827 | 29 | 54.45 | 0.004 |
| 2023 | CCL2 | European | 14736 | GCST90274822 | 2146 | 18.29 | 0.001 |
| 2023 | CCL8 | European | 14733 | GCST90274821 | 33 | 36.03 | 0.003 |
| 2023 | CCL7 | European | 11783 | GCST90274823 | 32 | 28.30 | 0.003 |
| 2023 | CCL13 | European | 14736 | GCST90274824 | 33 | 45.86 | 0.003 |
| 2023 | CD244 | European | 14735 | GCST90274771 | 35 | 33.39 | 0.002 |
| 2023 | NT-3 | European | 14744 | GCST90274829 | 34 | 22.60 | 0.002 |
| 2023 | NRTN | European | 11791 | GCST90274828 | 28 | 21.41 | 0.002 |
| 2023 | OSM | European | 14736 | GCST90274831 | 29 | 26.72 | 0.002 |
| 2023 | OPG | European | 14733 | GCST90274830 | 33 | 33.66 | 0.002 |
| 2023 | PD-L1 | European | 14736 | GCST90274832 | 33 | 25.16 | 0.002 |
| 2023 | EN-RAGE | European | 14743 | GCST90274786 | 30 | 27.50 | 0.002 |
| 2023 | SLAMF1 | European | 14734 | GCST90274835 | 43 | 30.38 | 0.002 |
| 2023 | SIRT2 | European | 14736 | GCST90274834 | 24 | 25.77 | 0.002 |
| 2023 | STAMPB | European | 14736 | GCST90274837 | 27 | 21.99 | 0.002 |
| 2023 | SCF | European | 14736 | GCST90274833 | 47 | 36.22 | 0.003 |
| 2023 | SULT1A1 | European | 11793 | GCST90274836 | 36 | 30.66 | 0.003 |
| 2023 | CD5 | European | 14735 | GCST90274773 | 35 | 30.30 | 0.002 |
| 2023 | CD6 | European | 14735 | GCST90274774 | 2081 | 17.89 | 0.001 |
| 2023 | TSLP | European | 11793 | GCST90274845 | 27 | 21.84 | 0.002 |
| 2023 | TNFB | European | 11792 | GCST90274840 | 2194 | 17.22 | 0.002 |
| 2023 | TRANCE | European | 14736 | GCST90274844 | 54 | 33.22 | 0.002 |
| 2023 | TRAIL | European | 14735 | GCST90274843 | 42 | 50.70 | 0.004 |
| 2023 | TGF-alpha | European | 14733 | GCST90274838 | 28 | 23.64 | 0.002 |
| 2023 | TWEAK | European | 11785 | GCST90274839 | 31 | 21.82 | 0.002 |
| 2023 | TNF | European | 14736 | GCST90274846 | 50 | 30.24 | 0.002 |
| 2023 | TNFSF14 | European | 11793 | GCST90274842 | 41 | 30.58 | 0.003 |
| 2023 | TNFRSF9 | European | 11784 | GCST90274841 | 37 | 24.91 | 0.002 |
| 2023 | uPA | European | 14734 | GCST90274847 | 44 | 30.37 | 0.002 |
| 2023 | VEGF_A | European | 14744 | GCST90274848 | 40 | 63.79 | 0.004 |

A	B

|   C |   D |
| --- | --- |

Figure1 Sensitivity analyses of the causal associations between 3-Phenylpropionylglycine and PD. (A) Scatter plot showing the SNP-specific associations between genetically predicted 3-Phenylpropionylglycine and PD risk. (B)Forest plot presenting the effect estimates of individual SNPs on PD risk.(C) Funnel plot indicating no evidence of heterogeneity across SNPs.(D) Leave-one-out analysis demonstrating that the causal estimates were not driven by any single SNP.

A	B

A	B

Figure2 Sensitivity analyses of the causal associations between 7-methylxanthine and PD. (A) Scatter plot showing the SNP-specific associations between genetically predicted 7-methylxanthine and PD risk. (B)Forest plot presenting the effect estimates of individual SNPs on PD risk.(C) Funnel plot indicating no evidence of heterogeneity across SNPs.(D) Leave-one-out analysis demonstrating that the causal estimates were not driven by any single SNP.

|   A | B | C |
| --- | --- | --- |

Figure3 Scatter plots of Mendelian randomization analyses for inflammatory proteins and PD risk. (A) Flt3L. (B) SULT1A1. (C) CCL13.

|   A |   B |   C |
| --- | --- | --- |

Figure4 Forest plots of Mendelian randomization analyses for inflammatory proteins and PD risk. (A) Flt3L. (B) SULT1A1. (C) CCL13.

|   A |   B |   C |
| --- | --- | --- |

Figure5 Funnel plots of Mendelian randomization analyses for inflammatory proteins and PD risk. (A) Flt3L. (B) SULT1A1. (C) CCL13.

|   A |   B |   C |
| --- | --- | --- |

Figure6 Leave-one-out (LOO) analysis for the association between inflammatory proteins and PD (A) Flt3L. (B) SULT1A1. (C) CCL13.
