# Supplementary material for: Exploring metabolic candidates and inflammatory mediation in Parkinson’s disease: a pilot integrative metabolomics and Mendelian randomization study
Source: PeerJ. 2026 Sep 28;14:e21670. doi: 10.7717/peerj.21670 (PMC13629568; doi:10.7717/peerj.21670)
Supplement: Supplementary file 2 [file peerj-14-21670-s002.zip › Raw data/2. Chromatogram/LYNC7-BPC-LCMS-neg.pdf]

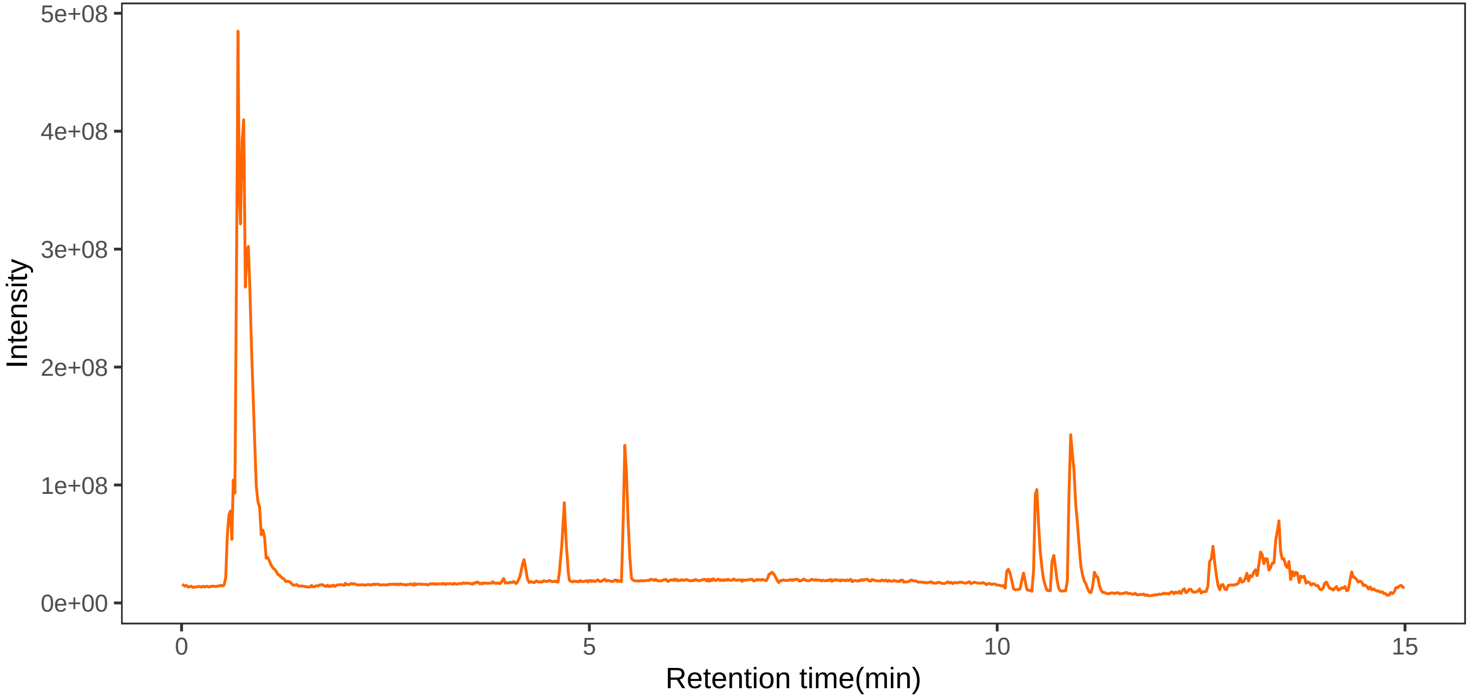

Intensity
5e+08
4e+08
3e+08
2e+08
1e+08
0e+00
0
5
10
15
Retention time(min)
