# Supplementary material for: Exploring metabolic candidates and inflammatory mediation in Parkinson’s disease: a pilot integrative metabolomics and Mendelian randomization study
Source: PeerJ. 2026 Sep 28;14:e21670. doi: 10.7717/peerj.21670 (PMC13629568; doi:10.7717/peerj.21670)
Supplement: Supplementary file 2 [file peerj-14-21670-s002.zip › Raw data/2. Chromatogram/P122-BPC-LCMS-pos.pdf]

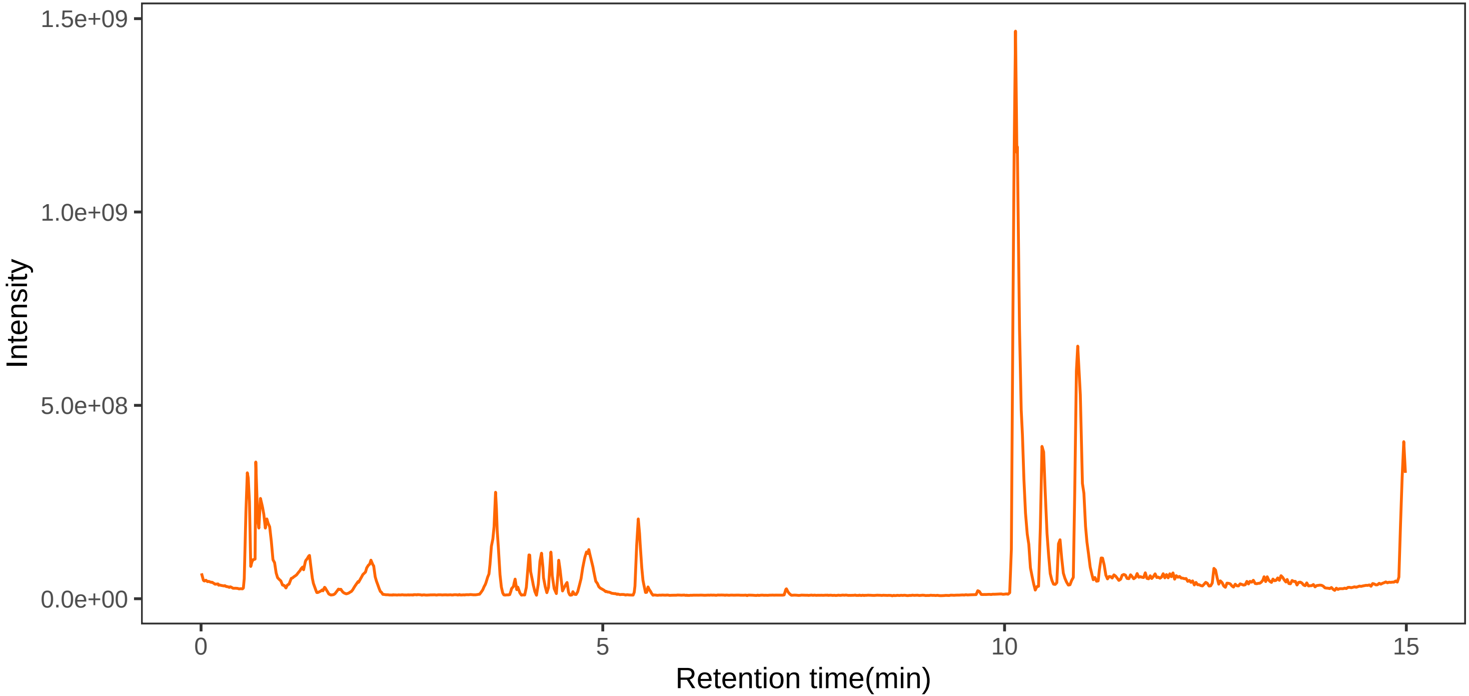

1.5e+09
1.0e+09
5.0e+08
0.0e+00
Intensity
0
5
10
15
Retention time(min)
